# Drought Stress-Mediated Transcriptome Profile Reveals NCED as a Key Player Modulating Drought Tolerance in *Populus davidiana*

**DOI:** 10.3389/fpls.2021.755539

**Published:** 2021-10-28

**Authors:** Sang-Uk Lee, Bong-Gyu Mun, Eun-Kyung Bae, Jae-Young Kim, Hyun-Ho Kim, Muhammad Shahid, Young-Im Choi, Adil Hussain, Byung-Wook Yun

**Affiliations:** ^1^School of Applied Biosciences, Kyungpook National University, Daegu, South Korea; ^2^Forest Microbiology Division, National Institute of Forest Science, Suwon-si, South Korea; ^3^Agriculture Research Institute, Mingora, Swat, Pakistan; ^4^Forest Biotechnology Division, National Institute of Forest Science, Suwon-si, South Korea; ^5^Department of Agriculture, Abdul Wali Khan University Mardan, Mardan, Pakistan

**Keywords:** NCED3, NCED1, drought, *Populus davidiana*, ABA, transcriptome

## Abstract

*Populus trichocarpa* has been studied as a model poplar species through biomolecular approaches and was the first tree species to be genome sequenced. In this study, we employed a high throughput RNA-sequencing (RNA-seq) mediated leaf transcriptome analysis to investigate the response of four different *Populus davidiana* cultivars to drought stress. Following the RNA-seq, we compared the transcriptome profiles and identified two differentially expressed genes (DEGs) with contrasting expression patterns in the drought-sensitive and tolerant groups, i.e., upregulated in the drought-tolerant *P. davidiana* groups but downregulated in the sensitive group. Both these genes encode a 9-cis-epoxycarotenoid dioxygenase (*NCED*), a key enzyme required for abscisic acid (ABA) biosynthesis. The high-performance liquid chromatography (HPLC) measurements showed a significantly higher ABA accumulation in the cultivars of the drought-tolerant group following dehydration. The Arabidopsis *nced3* loss-of-function mutants showed a significantly higher sensitivity to drought stress, ~90% of these plants died after 9 days of drought stress treatment. The real-time PCR analysis of several key genes indicated a strict regulation of drought stress at the transcriptional level in the *P. davidiana* drought-tolerant cultivars. The transgenic *P. davidiana* NCED3 overexpressing (OE) plants were significantly more tolerant to drought stress as compared with the NCED knock-down RNA interference (RNAi) lines. Further, the NCED OE plants accumulated a significantly higher quantity of ABA and exhibited strict regulation of drought stress at the transcriptional level. Furthermore, we identified several key differences in the amino acid sequence, predicted structure, and co-factor/ligand binding activity of NCED3 between drought-tolerant and susceptible *P. davidiana* cultivars. Here, we presented the first evidence of the significant role of *NCED* genes in regulating ABA-dependent drought stress responses in the forest tree *P. davidiana* and uncovered the molecular basis of *NCED3* evolution associated with increased drought tolerance.

## Introduction

Drought is an atmospheric condition and one of the most important abiotic stresses for plants, which has gradually become more severe, consistent, and ubiquitous, primarily because of global changes in weather patterns. Drought stress occurs when sufficient rain or irrigation water is not available at the right time for crop production. The production of many crops is now under threat due to chronic water shortages in many countries of the world. According to the global drought information system (www.drought.gov), *El Nino* continued to influence the global weather up to the end of February 2016. *El Nino* [which is the warm phase of the *El Nino* Southern Oscillation (ENSO)] events have been occurring for thousands of years, however, their significance has been realized only recently due to their greater impact on the global weather phenomena. It is thought that at least 30 *El Nino* events have occurred since the year 1900 with the strongest events occurring on 1982–83, 1997–98, and 2014–16 (http://www.cpc.ncep.noaa.gov/products/analysis_monitoring/ensostuff/ensoyears.shtml). “*El Nino* conditions” (duration 7–9 months) and “*El Nino* episodes” (duration longer than 9 months) are becoming more common now, increasing the global temperature. According to the National Oceanic and Atmospheric Administration (NOAA)—the USA and the National Aeronautics and Space Administration (NASA)—USA, February 2016 was the warmest February in the 137 years of the history of Earth whereas, February 2017 was the second warmest (https://climate.nasa.gov/news/2564/february-2017-was-second-warmest-february-on-record/). This global phenomenon is changing world weather by a tremendous magnitude leading to abnormal rainfall patterns ultimately causing drought stress.

Drought stress affects the overall plant growth, membrane integrity, chlorophyll and water content, cell turgor, stomatal movement, photosynthesis, respiration, translocation, ion, and nutrient uptake, and the production of phytohormones ultimately leading to the death of the plant. Since water is extremely essential for all biological activities inside plant cells/tissues, its shortage exerts multi-dimensional stress. This triggers the activation of a wide array of responses at the cellular level [reviewed by Kaur and Asthir (2017)] which ultimately determines plant survival. During the last decade, a plethora of published research investigated the genetic and molecular mechanisms underlying drought tolerance (Kaur and Asthir, 2017). The phytohormone abscisic acid (ABA) plays an important role in regulating drought stress tolerance in plants and has been used as an indicator while testing various plant species for drought tolerance. The production of ABA and reactive oxygen species (ROS) such as hydrogen peroxide (H_2_O_2_) are the inevitable consequences of drought stress in plants. The production of ABA in the roots and leaves is induced by drought stress which is then translocated throughout the plant shoot leading to stomatal closure (Mittler and Blumwald, 2015). Recently, Andres et al. (2014) showed that the apoplastic pH potentiated by the plant potassium uptake determines the stomatal aperture through the alkaline trapping of ABA in the xylem/apoplast increasing the ABA levels in the guard cells. ABA is not only important for drought stress tolerance, but it also plays an important role in other cellular processes including seed development and dormancy, germination, and plant growth.

Since its discovery, ABA production in plants has been the center of attention for many scientists. The ABA biosynthetic pathway in higher plants is now highly understood, thanks to a wide range of genetic and biochemical studies. The study/analysis of ABA deficiency has been instrumental in revealing the intricate details of ABA biosynthesis and downstream signal transduction. These studies revealed an “indirect” ABA biosynthetic pathway through the breakdown of a C_40_ carotenoid precursor, followed by the conversion of xanthoxin to ABA [see Figure 1 of Xiong and Zhu (2003)]. Through a step-wise reaction, the zeaxanthin epoxidase (ZEP) catalyzes the epoxidation of antheraxanthin and zeaxanthin to violaxanthin in the plastids. Violaxanthin is later structurally modified to form 9-*cis*-neoxanthin which is then cleaved by the 9-*cis*-epoxycarotenoid dioxygenase (NCED) yielding xanthoxin (Schwartz et al., 1997) which, after the transportation to the cytosol, is converted into ABA through *ABA2* (Rook et al., 2001). The genetic evidence for the role of the *NCED* gene in ABA biosynthesis came from a study involving a maize *vp14* mutant (Tan et al., 1997). Later, Thompson et al. (2000b) also showed the involvement of *LeNCED1* in ABA biosynthesis as the ectopic expression of this gene significantly increased the ABA levels in tomato seeds and extended seed dormancy. The *NCED* is a multi-gene family in several plant species such as maize, avocado, tomato, and Arabidopsis, as sequence homology-based Basic Local Alignment Search Tool (BLAST) analysis shows the existence of the large gene family in plants (Chernys and Zeevaart, 2000). Drought stress induces the expression of *NCED* genes in maize (Tan et al., 1997), tomatoes (Burbidge et al., 1999), cowpeas (Iuchi et al., 2000), Arabidopsis (Iuchi et al., 2001), beans (Qin and Zeevaart, 1999), and avocadoes (Chernys and Zeevaart, 2000). The induction of *NCED* genes in response to water shortage is fairly quick as a significant increase in the *NCED* transcript accumulation has been observed within 15 to 30 min of dehydration treatment or leaf detachment (Qin and Zeevaart, 1999; Thompson et al., 2000a). In this study, we screened different *Populus davidiana* cultivars for their response to drought stress, eventually isolating two drought-resistant and two drought susceptible cultivars. Furthermore, through RNA-sequencing (RNA-seq) the mediated transcriptome profiling following dehydration, we show for the first time that *NCED* is a key player modulating drought stress responses in this species.

**Figure 1:**
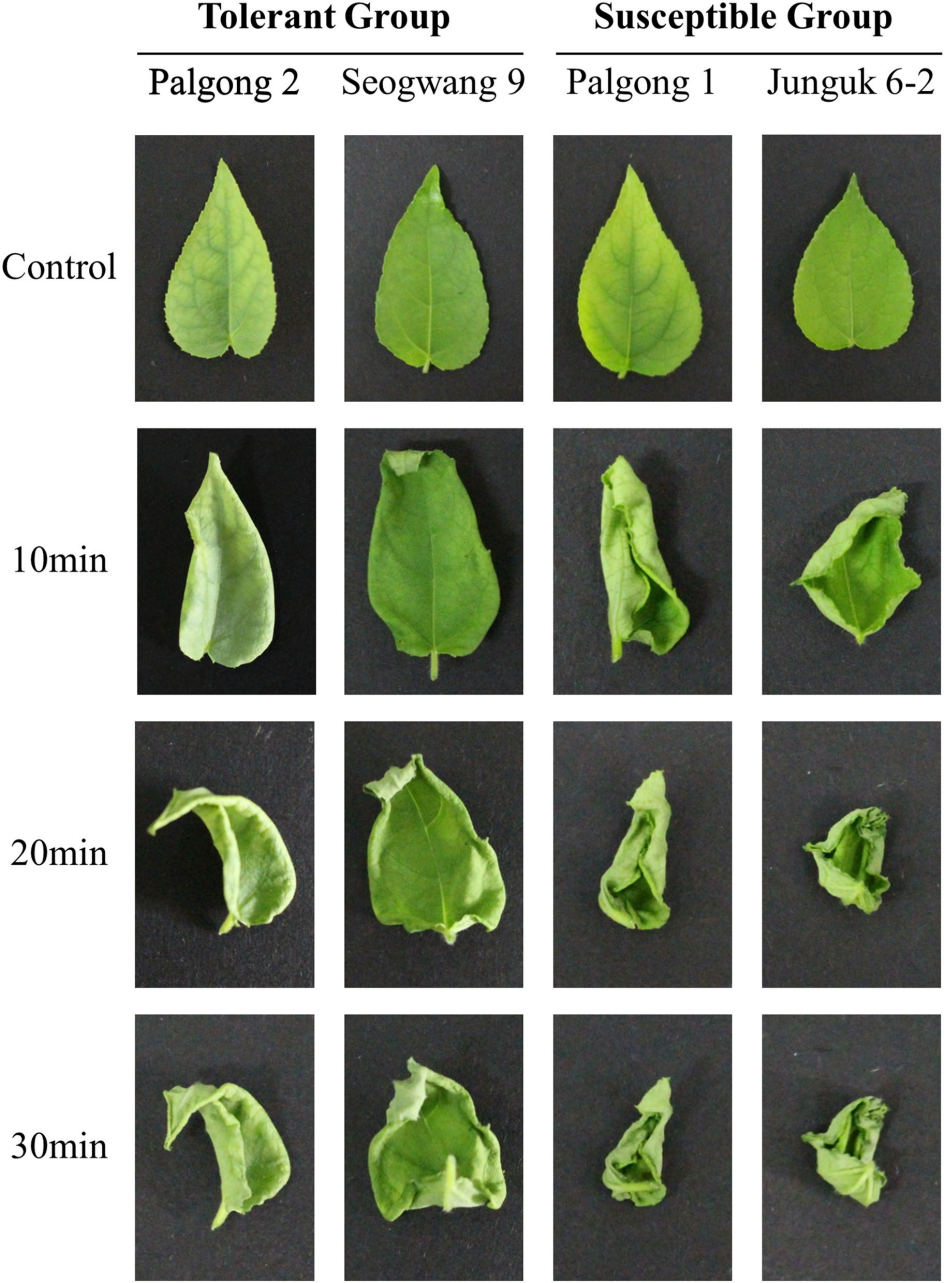
The response of drought-resistant and susceptible *Populus davidiana* cultivars to dehydration. The leaves were detached and allowed to sit for 30 min at room temperature. The leaves of Palgong 1 and Junguk 6–2 showed severe curling and rolling which is a characteristic of moisture stress after 10, 20, and 30 min as compared with the leaves of Palgong 2 and Seogwang 9, proving the significantly tolerant response of Palgong 2 and Seogwang 9 to dehydration. “Control” leaves were detached and pictures were taken immediately without the stress treatment.

## Materials and Methods

### Plant Materials and Growth Conditions

Four different cultivars of *P. davidiana* were acquired from the National Institute of Forest Science (NIFoS), Suwon, Korea. The cultivars were divided into two different groups (drought tolerant group and drought susceptible group) based upon their overall growth response to water shortage/drought stress. The tolerant group contained two cultivars named Palgong 2 and Seogwang 9, whereas the susceptible group contained two cultivars named Palgong 1 and Junguk 6–2. The seeds of the four *P. davidiana* cultivars were surface-sterilized with H_2_O_2_ and washed five times with sterile distilled water and germinated on a half Murashige and Skoog (MS) medium. After 4 weeks, the plantlets were transferred on full-strength MS salts and vitamins supplemented with 3% Sucrose, 0.27% Gelrite, 0.5 ppm NAA at pH = 5.8 for organogenesis and incubated at 16 h/8 h light–dark cycle at 22–23°C for 4 weeks.

### Dehydration Stress Treatment

Five plants from each cultivar of each group were selected. For the dehydration stress treatment, whole 8-week-old plants were carefully uprooted from the MS medium and allowed to stand/dry at room temperature and humidity on a dry filter paper for up to 30 min.

### Stomatal Structure Analysis

Stomatal conductance plays a crucial role in regulating plant responses to dehydration. To determine whether *PdNCED3* is involved in stomatal regulation, we investigated the stomatal structure of all the four poplar cultivars after 30 min of dehydration. Furthermore, the same investigations were performed for the wild type (WT), overexpressing (OE), and RNA interference (RNAi) lines under control and dehydration stress. For this purpose, we collected over 10 leaves each from five plantlets of each cultivar or line. A thin layer of transparent nail polish was applied to the bottom of the leaves and allowed to dry for several minutes. Transparent scotch tape was applied to the dried nail polish and carefully pressed starting from one side to remove the air bubbles. The tape was carefully removed from the leaves. This imprints the stomata and the surrounding cells onto the tape. The tape was then mounted on a microscopic slide. The abaxial leaf surface of each sample was observed this way using an Axioplan 2 imaging microscope (Axioplan 2 imaging; Carl Zeiss, Jena, Germany). Pictures were taken and analyzed using ImageJ (Schneider et al., 2012) to measure the stomatal apertures as described by Wu et al. (2017).

### Weight Loss Measurement

The plants were grown as described above and were subjected to dehydration stress at 8-weeks old. The plants were uprooted and kept on a dry filter paper at room temperature. The weight of all the plants was measured 1, 2, 3, and 4 h after uprooting to calculate the percent loss in weight.

### DAB Staining

As described above, the production of ROS such as H_2_O_2_ is one of the inevitable consequences of drought/water stress. For the quantification of drought-induced H_2_O_2_ accumulation, we performed 3,3′-diaminobenzidine (DAB) staining of plant leaves after every 2 min of uprooting for up to 14 min. The DAB staining was performed on three leaves of each cultivar from both the groups as described by Daudi and O'brien (2012). Images of the stained leaves were taken with a Canon EOS700D SLR camera (Canon, Ota City, Tokyo, Japan) and were edited in Adobe Photoshop CS6 (Adobe, San Jose, California, United States) as described by Imran et al. (2016). Briefly, the pictures were imported to Photoshop and the intensity of the DAB stain was measured inside a fixed rectangular shape (drawn through the marquee tool) using the luminosity tool. The measurement was performed at a minimum of three different places per picture.

### ABA Quantification

After the dehydration stress treatments, the whole plants were stored in liquid nitrogen and freeze-dried using a freeze dryer (ISE Bondiro, South Korea). The freeze-dried samples were ground to a fine powder and the endogenous ABA content was measured as described by Kamboj et al. (1999). Briefly, the samples were treated with 10 ml of an extraction solution containing 95% of isopropanol, 5% glacial acetic acid, and 100 ng of [(±)-3,5,5,7,7,7-d6]-ABA. After collecting the aqueous phase, the extracts were evaporated and the residues were dissolved in 4 ml of 1N sodium hydroxide solution. The suspension was again evaporated and the residues were washed three times with 3 ml of methylene chloride to remove the lipophilic material. The aqueous phase was brought to approximately pH 3 with N hydrochloric acid and partitioned three times with ethyl acetate (EtOAc). The EtOAc extracts were combined and evaporated. The dried residue was dissolved in a phosphate buffer (pH8) and mixed with 1 g of polyvinylpolypyrrolidone (PVPP) powder on a shaker for 1 h. The extracts were added into reaction vessels (1 ml) and were methylated by adding diazomethane for the GC/MS-SIM (6890N network GC system, 5,793 networks mass-selective detector; Agilent Technologies, USA) analysis and quantification using the Lab-Base (ThermoQuest, Manchester, UK) software.

### RNA Extraction and RNA-Sequencing

RNA extraction and sequencing were performed as described by Hussain et al. (2016). Briefly, the samples were collected after 10 min of the treatment. The finely ground homogenized samples were lysed in a TRIzol reagent (Invitrogen, Carlsbad, CA, USA), and the total RNA was extracted from each sample. For the sequencing, the complementary DNA (cDNA) was synthesized from 2 μg of the total RNA, using a DiaStar^TM^ RT Kit (Solgent, Daejeon, South Korea). The RNA-seq was performed with the Hiseq2500 sequencer (Illumina, San Diego, California, United States). The raw reads were processed to identify the high-quality reads with a threshold level of Q20 > 40% and the reads with more than 10% ambiguous bases or with Q20 < 40% were removed. The high-quality reads were compared with the reference genome of *P. trichocarpa* in Ensembl (Flicek et al., 2014) and aligned using TopHat (Trapnell et al., 2009) with default values. The gene expression levels were calculated using Cufflinks (Trapnell et al., 2012). The DEGs were identified at *p* < 0.05 using Cuffdiff (Trapnell et al., 2012). The data were submitted to the Gene Expression Omnibus (GEO) at the National Center for Biotechnology Information (NCBI) (http://www.ncbi.nlm.nih.gov/) with accession numbers GSE98170 (Junguk 6–2), GES98172 (Palgong 1), GES98173 (Palgong 2), and GES98175 (Seogwang 9).

### MapMan Analysis

Large-scale genome sequencing projects such as RNA-seq usually yield data comprising of several thousand genes and transcripts. Handling such a large amount of data is often very challenging and it is often difficult to extract meaningful information at the functional level. To get around this problem, we analyzed the transcriptomic data through MapMan (version 3.6.0RC1) as described in Hussain et al. (2016). MapMan is an omics data analysis software that allows the visualization of the DEGs at functional and pathway levels (Thimm et al., 2004). For this purpose, the list of all the DEGs obtained from each cultivar of each group was uploaded to MapMan and mapped against the poplar database “Ptrichocarpa V3.0_210.” The involvement of DEGs in various pathways and cellular processes was determined by applying the respective BIN and Sub-BIN numbers on custom-made images uploaded to MapMan.

### Quantitative/Real-Time PCR Analysis

The validation of the RNA-seq data was carried out using the real-time quantitative reverse transcription PCR (qRT-PCR) analysis. For this purpose, the genes related to the ABA-pathway were chosen. The RNA was extracted using a Trizol reagent (Invitrogen, USA) and the cDNA was synthesized from 2 μg of the total RNA using a DiaStar^TM^ RT Kit (SolGent, Korea). A two-step PCR was performed using the primers given in Supplementary Table 1 in the Eco^TM^ Real-Time PCR machine (Illumina, USA). The PCR reaction was performed in a total volume of 20 μl using 2 × QuantiSpeed SYBR Green Kit (PhileKorea, Seoul, Korea) with 10 nM of each primer and 100 ng of the template DNA under the following conditions: polymerase activation at 95°C for 5 s, 18 concurrent annealing, and extension steps at 60°C each for 30 s. Poplar actin (*Potri.001G309500*) was used as a comparative control. A no-template reaction was also used as a control. The qRT-PCR was performed using at least three independent biological replicates and three technical replicates of each biological replicate.

### Sequence Alignment and 3D Structure Prediction

The comparison of the *NCED* sequence of *P. davidiana* with that of *P. trichocarpa* was carried out using the Integrative Genomics Viewer (IGV 2.3.72) program (Robinson et al., 2011). The amino acid sequences of two *NCED* genes of *P. davidiana* were submitted to the I-TASSER server for the protein model prediction. The I-TASSER produced one to five models for each of the poplar sequences. Among these, the model with the highest confidence score (C-score) was selected and analyzed using the PyMOL software (LLC, http://www.pymol.org/).

### Vector Construction for NCED3 Overexpression and Knock-Down

The *Populus davidiana* overexpression (OE) and RNAi transgenic lines were generated using the Gateway (Thermofisher Scientific, Waltham, Massachusetts, United States) system as described in **Figures 8A,B**. The full coding DNA sequence of *PdNCED3* (*Potri.011G112400*) for the OE DNA construct and a 200 bp segment (starting from the start codon) for the RNAi DNA construct was amplified using a proof-reading polymerase enzyme for one-step cloning into PCR^®^8/GW/TOPO plasmid and transformed into TOP10 competent cells according to the instructions of the manufacturer and plated on Luria Broth (LB)-agar plates containing 50 mg/L of spectinomycin. The successfully transformed colonies were genotyped through PCR to confirm the presence of inserts. Plasmids were extracted and the sequences of inserts were confirmed using M13 universal primers. The sequences of primers used are given in Supplementary Table 1.

Afterward, the inserts with the correct sequences were transferred to the destination vectors via LR reaction according to the manufacturer's instruction. The OE DNA construct was transferred to the pK2WG7 destination vector whereas, the RNAi construct was transferred to the pK7GWIWG2(1) destination vector. The recombinant vectors were transformed into DH5α competent cells and plated on LB-agar plates containing 50 mg/L of Kanamycin (pK2WG7) and 50 mg/L of Kanamycin [pK7GWIWG2(1)]. The successfully transformed colonies were genotyped using PCR as described above to confirm the presence of the inserts. The destination vectors containing the OE and RNAi constructs were transformed into *Agrobacterium tumefaciens* strain GV3101.

### Plant Transformation *via* Tissue Culture

The *Agrobacterium tumefaciens* containing the OE and RNAi constructs were cultured in LB at 28°C in a shaking incubator at 150 rpm. After 48 h, 5 ml of this culture was added to 50 ml of a fresh LB culture medium and incubated again for 24 h. The bacteria were harvested by centrifugation at 3,000 rpm at 4°C for 15 min. The bacterial cells were re-suspended in a 25 ml saline solution on ice. The stems of hybrid poplar (*P. alba* × *P. glandulosa* cv. Bonghwa) cultivated for 1 month in a root-induction medium were cut into 5 mm tissue disks. The cut stem tissues were immersed in 5 ml of 0.85% sodium chloride (NaCl). The stem tissues were dipped in a bacterial saline suspension for 15 min after adding 25 ml of acetosyringone (10 mg/ml). The cut stem tissues were then taken out of the bacterial saline and gently rinsed in a sterile saline solution. The process of inoculation and rinsing was repeated two times. The inoculated stem tissues were then cultured in a callus induction medium without antibiotics and incubated for 2 days and then transferred to a fresh callus induction medium supplemented with 50 mg/ml of kanamycin (for OE construct) and 50 mg/ml of kanamycin (for RNAi construct) for 2 weeks. The resulting calli were transferred to a fresh media after every 2 weeks in the same way. The successful calli were formed within 4 weeks of culture. The calli of 2 to 3 mm size were transferred to a stem-induction medium in the presence of kanamycin as described above. Afterward, the plants were transferred to a root induction medium (Supplementary Figure 7).

### Statistical Analysis

All the experiments were replicated at least three times. Data were collected at regular intervals. The means and SDs were calculated in Microsoft Excel. The means were separated for significant differences using Student's *T*-test at a 5% level of confidence.

## Results

### Isolation of Drought Resistant and Susceptible *P. davidiana* Cultivars

**Figure 2:**
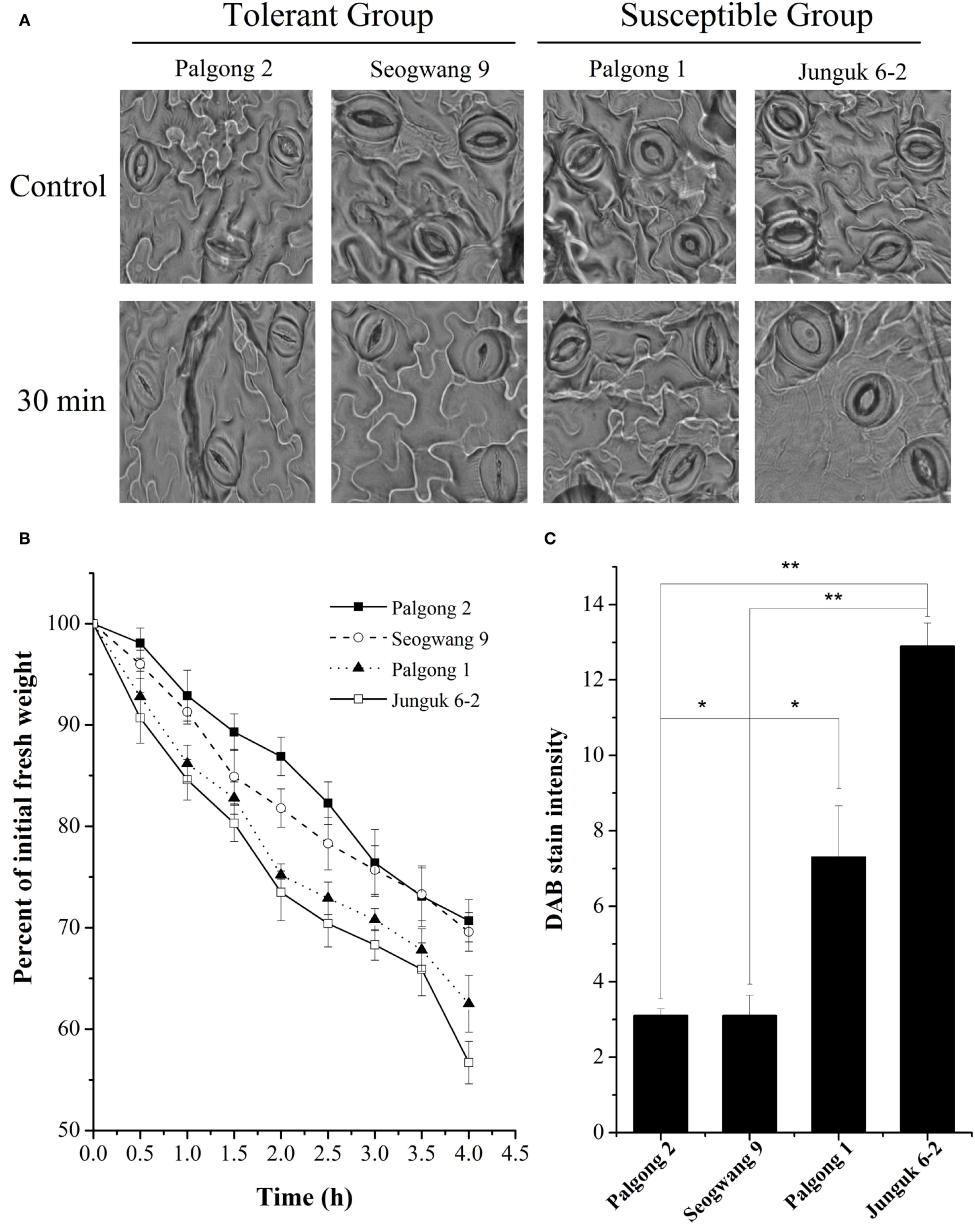
Stomatal conductance in response to dehydration. Scanning electron micrographs showed the stomatal aperture of the drought-tolerant and susceptible plants before and after 30 min of dehydration stress **(A)**. Percent loss in fresh weight after 4 h of dehydration stress **(B)**. Quantification of hydrogen peroxide (H_2_O_2_) released after 30 min of dehydration stress measured by diaminobenzidine (DAB) staining **(C)**. Each data point shows the mean of at least three replicates. Error bars represent SD. Significant differences at *P* < 0.05 are indicated by ^*^. Significant differences at *P* < 0.01 are indicated by **.

While screening different *P. davidiana* cultivars for their response to drought stress, we identified two drought-resistant cultivars (Palgong 2 and Seogwang 9) and two drought susceptible cultivars (Palgong 1 and Junguk 6–2). According to the field and screen house-based drought stress experiments, the detached leaves of the cultivars Palgong 1 and Junguk 6–2 showed severe curling and leaf rolling after 10, 20, and 30 min of detachment which are characteristic symptoms of moisture stress (Figure 1). On the other hand, Palgong 2 and Seogwang 9 exhibited significantly less and delayed curling and leaf rolling. The dehydration resistance shown by Palgong 2 and Seogwang 9 was supported by the stomatal closure after 30 min (Figure 2A). This was also accompanied by a significantly higher linear loss of fresh weight by the susceptible cultivars Palgong 1 and Junguk 6–2, due to a high rate of evapotranspiration from the leaves. The leaves of these cultivars lost 37 and 43% of their fresh weight, respectively within the first 4 h of the treatment (Figure 2B). As the shortage of moisture/water inside plant tissues is characterized by high levels of H_2_O_2_ in leaf tissues, we quantified the amount of H_2_O_2_ produced in the leaves *via* DAB staining, following 30 min of dehydration. The results supported our previous findings as Palgong 1 and Junguk 6–2 showed significantly higher DAB stain intensity as compared with the tolerant cultivars indicating a much higher level of H_2_O_2_ production in the leaves of these plants (Figure 2C). The ability of plants to resist water loss is also reflected by their ability to regulate stomatal movement under stress. We subjected the four cultivars to 30 min of dehydration following detachment and then observed their stomata through an electron microscope. The stomata of Palgong 1 and Junguk 6–2 (susceptible cultivars) had a significantly higher aperture as compared to the drought-tolerant cultivars Palgong 2 and Seogwang 9 (Supplementary Figure 6A).

### Transcriptome Analysis

**Figure 3:**
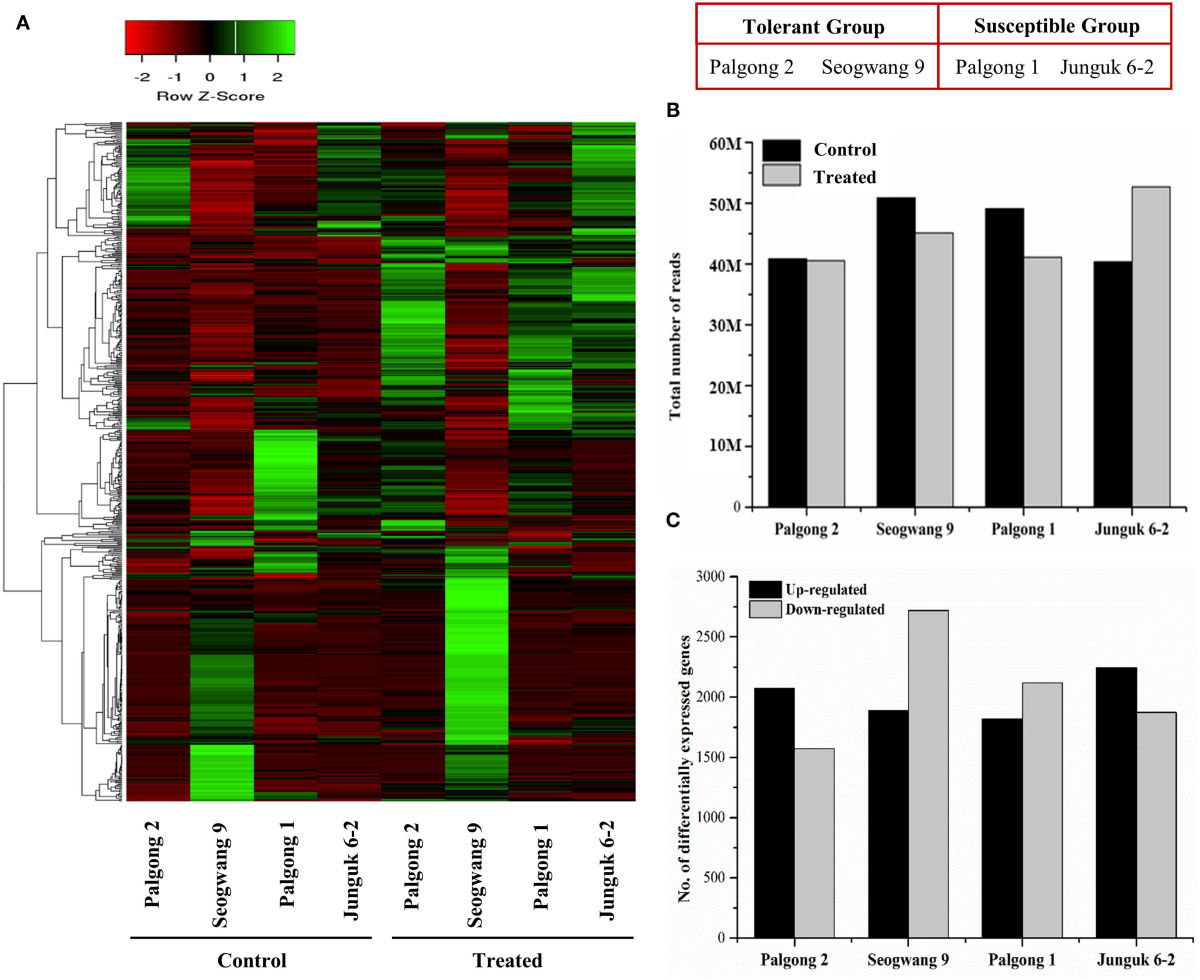
Transcriptome analysis of drought-resistant and susceptible *Populus davidiana* cultivars following dehydration. Heat map showing the signal intensities of the differentially expressed genes (DEGs) in the control and treated samples of each cultivar after 10 min **(A)**. An average of 45.34 and 44.89 million reads were obtained from the stressed and control samples, respectively **(B)**. At a Q-value of 0.05, an average of 2,008 upregulated and 2,071 downregulated genes were identified in all the cultivars **(C)**.

The analysis of the transcriptomic data showed highly significant changes in expression at a cutoff Q value of 0.05 (Figure 3A). An average of 45.34 million reads was obtained from the stressed plants compared with 44.89 million reads (Figure 3B; Supplementary Table 2) from the control samples. Up to 55.36% of these reads were successfully mapped onto the *P. trichocarpa* reference genome. An average of 27,501 genes and 98,321 transcripts in the control samples and an average of 27,623 genes and 98,575 transcripts in the treated samples were identified (Supplementary Table 2). Further, a total of 16,318 DEGs were obtained following the stress treatment (Figure 3C). Among these, the drought-tolerant cultivars, Palgong 2 and Seogwang 9, showed an increase in the expression of 2,076 and 1,891 genes and a reduction in the expression of 1,573 and 2,719 genes, respectively. On the other hand, the drought susceptible clones, Palgong 1 and Junguk 6–2, yielded 1,822 and 2,244 upregulated genes along with 2,119 and 1,874 downregulated genes, respectively (Figure 3C). Interestingly, two genes that encode the NCED protein, *PdNCED1* (*Potri.001G393800*) and *PdNCED3* (*Potri.011G112400*), were upregulated in the drought-tolerant cultivars Palgong 2 and Seogwang 9 by more than 5 and seven folds, respectively, but significantly downregulated in the drought-susceptible cultivars (**Figure 7**).

### Regulation of Drought Stress Tolerance in *P. davidiana*

All the DEGs were analyzed to identify specific drought-related genes with statistically significant and at least two-fold change in their expression, among the drought-tolerant and drought-susceptible plants. The results showed that both the drought-tolerant cultivars expressed at least six drought-specific genes with the same expression patterns that were not present in the plants of the drought susceptible group (Supplementary Figure 1). Furthermore, there were at least three drought-specific genes that had contrasting expression patterns between the tolerant and susceptible groups, i.e., these were downregulated in the drought-tolerant plants but upregulated in the drought susceptible plants (Supplementary Figure 1) indicating their negative regulatory role in drought tolerance. These DEGs included *Potri.001g131400.3, Potri.005g226000.4*, and *Potri.011g009900.1*. All three DEGs encode early-responsive to dehydration stress (ERD3) proteins.

**Figure 4:**
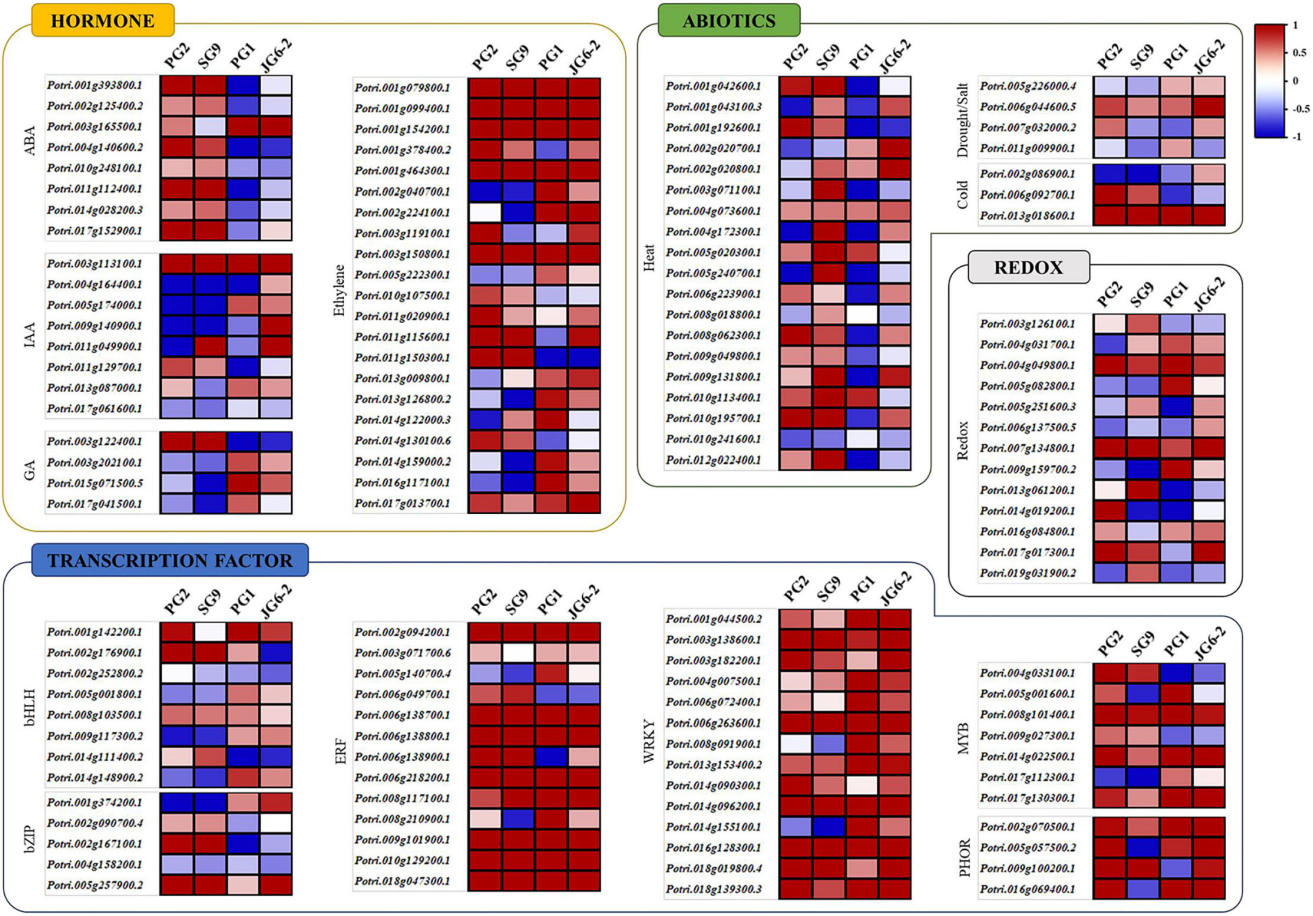
MapMan analysis of dehydration-responsive *P. davidiana* DEGs. Differentially expressed genes with statistically significant and at least two-fold change in expression following the treatment were analyzed using MapMan. Results showed DEGs involved in abiotic stress responses, heat stress regulation, signaling of developmental hormones such as IAA, ABA, Ethylene, GA and defense hormones JA and SA, redox regulation, and those encoding important transcription factors such as WRKY, ERF, bZIP, MYB, and bHLH.

For a further detailed analysis of the transcriptome profiles of drought-tolerant and susceptible cultivars, we analyzed the transcriptomic data through MapMan (Thimm et al., 2004). For this purpose, we chose genes with statistically significant and at least a two-fold change in their expression. The results showed that the genes belonging to a plethora of important physiological processes involved in abiotic stress responses were differentially expressed in the drought-tolerant and susceptible clones (Figure 4). These include more than 30 genes responsible for heat stress regulation, with differential expression patterns between the resistant and susceptible cultivars. Other groups with differential expression patterns among resistant and susceptible poplar plants included genes involved in the signaling of developmental hormones such as ABA, IAA, GA, and Ethylene. Drought and other abiotic stresses are often accompanied by a concomitant change in the cellular redox signature. We found several groups of DEGs involved in redox signaling such as multiple genes encoding thioredoxin, peroxiredoxin, glutaredoxin, catalase, and dismutase genes (Figure 4). The MapMan analysis also showed multiple DEGs encoding important transcription factors such as WRKY, ERF, bZIP, MYB, and bHLH that have a well-known role in abiotic stress responses.

**Figure 5:**
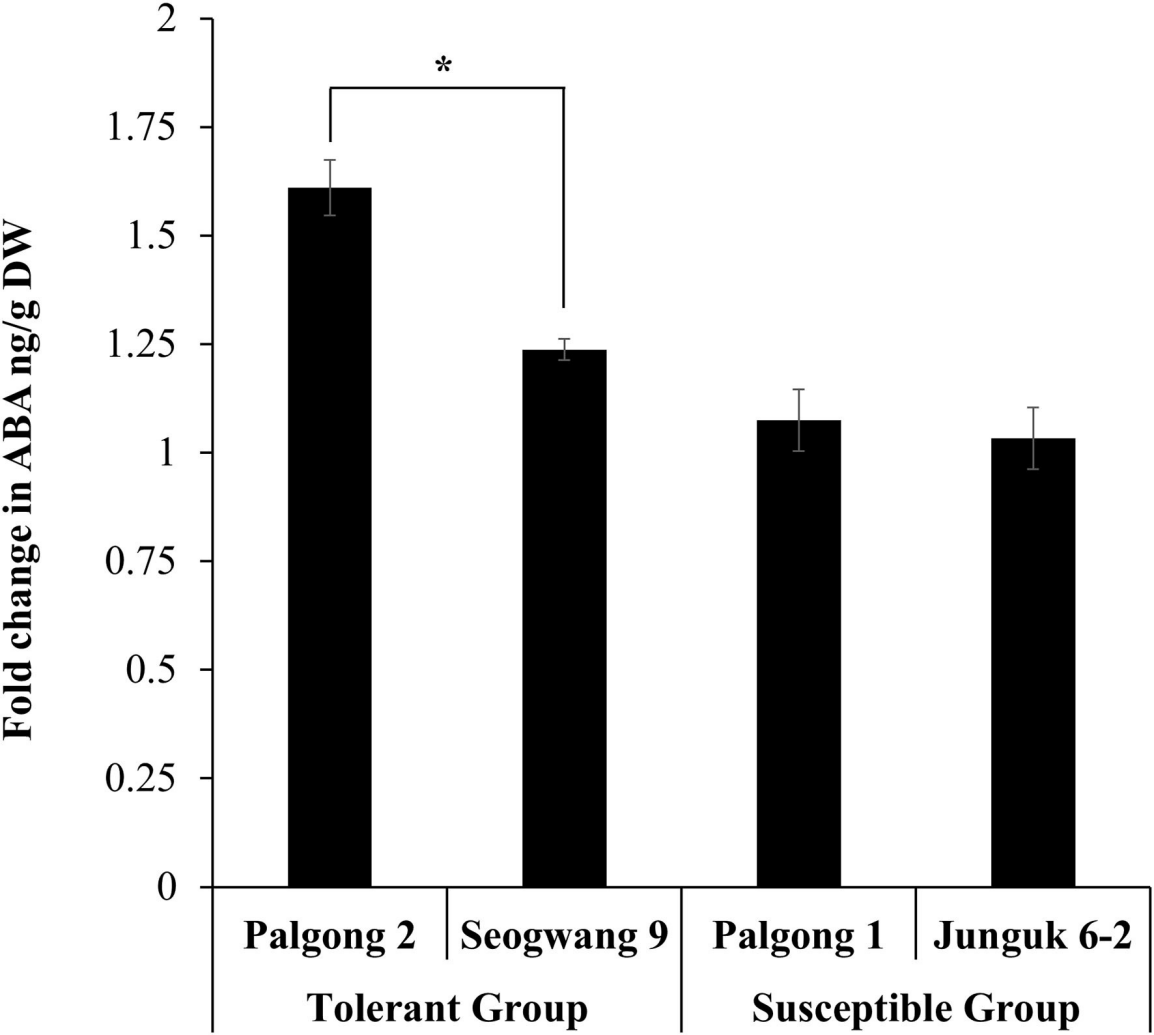
ABA quantification in drought-tolerant and susceptible *P. davidiana* cultivars following dehydration stress. The abscisic acid (ABA) measurement results showed a significantly higher increase in the ABA concentration of the leaves of the drought-tolerant *P. davidiana* cultivars Palgong 2 and Seogwang 9 after 30 min of dehydration stress as compared with the drought susceptible cultivars. Each data point represents the mean of at least three replications. Error bars represent SD. Significant differences at *P* < 0.05 are indicated by asterisks (*).

Genes encoding the NCED proteins (that catalyze the oxidative cleavage of carotenoids to synthesize ABA) have been shown to play a key role in the ABA-dependent drought tolerance in different plants (Schwartz et al., 1997; Tan et al., 1997; Burbidge et al., 1999; Qin and Zeevaart, 1999; Chernys and Zeevaart, 2000; Iuchi et al., 2000, 2001; Thompson et al., 2000a,b; Rook et al., 2001). We wondered if the response of drought-tolerant and susceptible plants used in this study was related to the ABA content in these plants. As expected, the ABA measurement results showed a significantly higher increase in the ABA content in Palgong 2 and Seogwang 9 poplar after 30 min of the stress treatment as compared with the plants of the drought susceptible group (Figure 5). These results were parallel to the transcriptomic expression patterns of *NCED* in these cultivars.

### NCED Regulates Drought Stress in *Arabidopsis thaliana*

**Figure 6:**
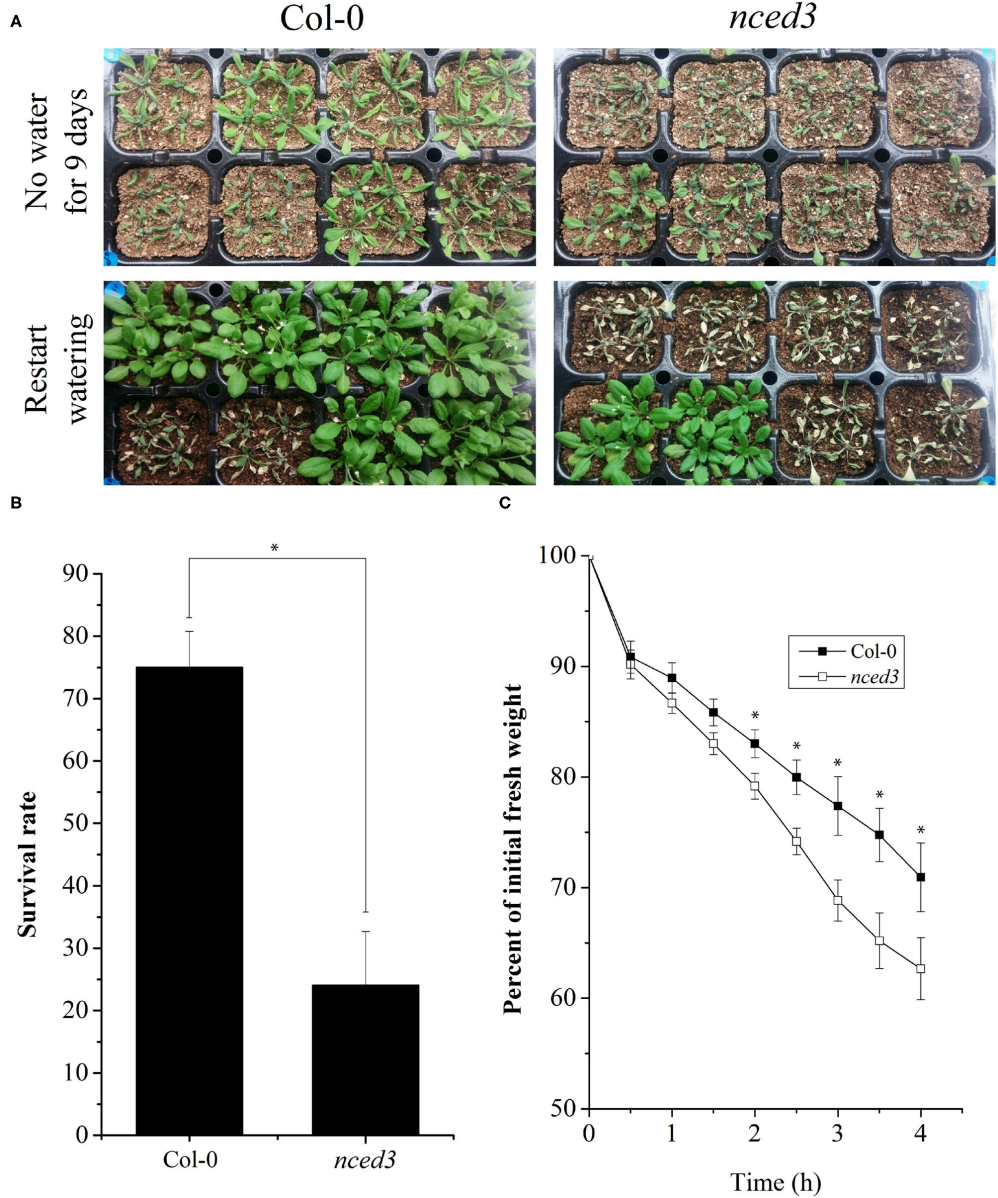
Role of NCED in drought stress responses of *Arabidopsis thaliana*. Arabidopsis *nced3* (*AT3G14440*) loss of function mutant plants were analyzed for their response to drought conditions. After 9 days of drought stress, 75% of the *nced3* plants died and could not recover with re-watering whereas, 75% of the wild type (WT) plants recovered **(A,B)**. Similarly, a significantly higher weight loss of the *nced3* plants occurred as a result of dehydration stress as compared with WT plants **(C)**. Each data point in the graphs represents the mean of at least three replications. Error bars represent SD. Significant differences between means at *P* < 0.05 are indicated by asterisks (*).

To further elucidate the functional role of *NCED* in regulating drought stress, we evaluated *Arabidopsis nced3* (*AT3G14440*) for the loss of function mutant plants for their response to drought conditions. Results showed that 75% of the *nced3* plants died after 9 days of continuous drought stress whereas, 75% of the WT plants recovered when the irrigation was re-started after 9 days (Figures 6A,B). This was also accompanied by a significantly higher weight loss of the *nced3* plants as compared to WT plants (Figure 6C).

### Validation of RNA-Seq Through ABA Pathway Genes

**Figure 7:**
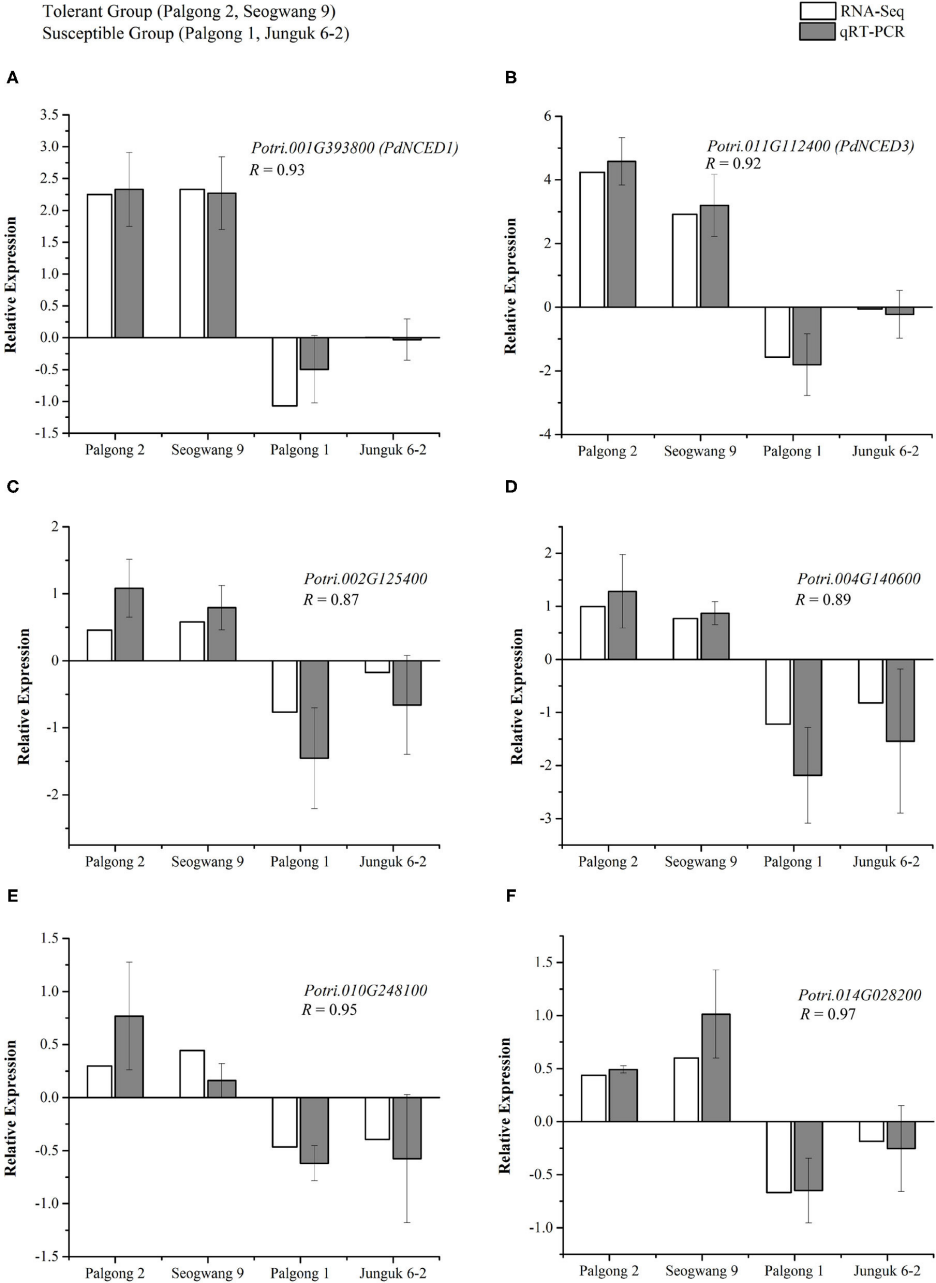
Validation of the expression patterns of drought-specific genes in *P. davidiana* following dehydration stress through qPCR. The two drought-responsive *NCED* genes; *PdNCED1* (*Potri.001G393800*) and *PdNCED3* (*Potri.011G112400*) were upregulated in the drought-tolerant cultivars Palgong2 and Seogwang 9 by more than five and seven folds respectively but downregulated in the drought-sensitive cultivars by two and 2.9 folds, respectively **(A,B)**. Four different ABA-responsive element (AREB) encoding genes (*Potri.002G125400, Potri.004G140600, Potri.010G248100*, and *Potri.014G028200*) were upregulated in the drought-tolerant cultivars but downregulated in the drought-sensitive cultivar **(C–F)**. As indicated by the significant correlation coefficients (R), RNA-sequencing (RNA-seq) and real-time quantitative reverse transcription PCR (qRT-PCR) results are highly comparable with each other. Each data point represents the mean of at least three replicates. Error bars indicate the SD.

To validate the expression patterns of the DEGs observed in the transcriptome, we performed real-time PCR analysis for six different genes following the dehydration stress. As described above, the expression patterns of the two drought-responsive *NCED* genes that are known to regulate ABA biosynthesis and signaling were very unique i.e., both the *PdNCED1* and *PdNCED3* were upregulated in the drought-tolerant cultivars Palgong 2 and Seogwang 9 by more than five- and seven-fold, respectively, but downregulated in the drought-sensitive plants by two- and 2.9-fold, respectively. The same expression patterns were produced by the qRT-PCR analysis (Figures 7A,B). As described earlier, members of the *NCED* gene family have been shown to positively regulate drought stress in plants in an ABA-dependent manner and further investigation showed four different ABA-responsive elements (AREB) genes (*Potri.002G125400, Potri.004G140600, Potri.010G248100*, and *Potri.014G028200*) to be upregulated in the drought-tolerant clones but downregulated in the drought-sensitive clones (Figures 7C–F). This indicates strict regulation of drought stress responses in these plants at the transcriptional level.

### *NCED3* Transgenic Poplar Plants Show Perturbed Drought Stress Responses

**Figure 8:**
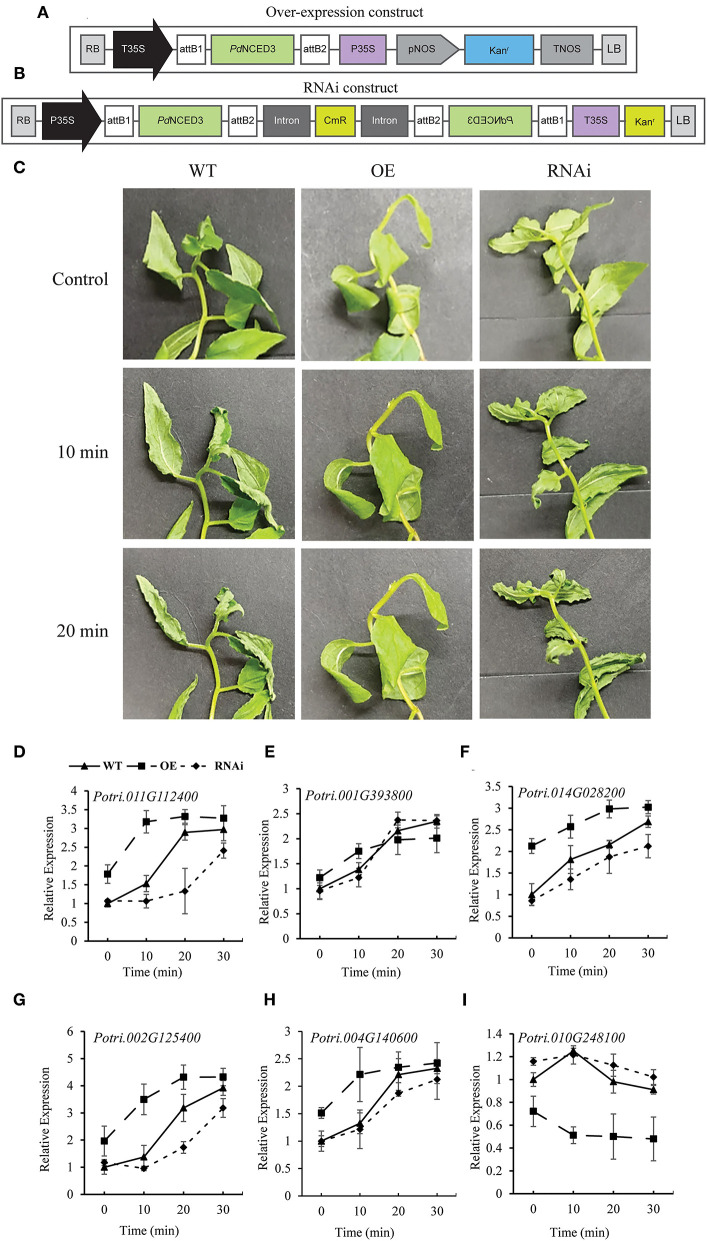
DNA constructs for generating transgenic lines and the response of transgenic lines to dehydration. The DNA constructs for generating NCED3 overexpression (OE) **(A)** and knock-down RNA interference (RNAi) lines **(B)**. The leaves from the OE and RNAi transgenic lines were detached and allowed to sit for up to 20 min at room temperature **(C)**. qRT-PCR analysis showing the expression of *NCED3* **(D)**, *NCED1* **(E)**, and multiple transcription factor genes involved in ABA-dependent drought stress responses **(F–I)**. Each data point in the graphs represents the mean of at least three replicates. Error bars indicate SD.

To further elucidate the role of NCED in the drought tolerance of *P. davidiana*, we generated transgenic NCED3 OE and RNAi lines using DNA constructs (Figures 8A,B). Generating OE and RNAi lines usually results in multiple lines. However, most often, different transgenic lines show different levels of expression of the transgene. Some OE lines show significantly high expression whereas others show only a small increase in expression of the target gene as compared with that in the WT lines. Similarly, some RNAi lines exhibit only a small reduction in the expression whereas, others may show a significant reduction in expression of the target gene. As far as we have observed in our laboratory, the transgenic lines with the expression profile of the transgene at the extremes (i.e., either the lowest or highest changes in expression compared with WT lines) do not often yield promising and reproducible results for all the parameters under study. In such situations, one may have to choose the lines with optimum changes in the expression of the target gene or lines that give promising and reproducible results in multiple experiments or for all or most of the parameters under study. We got several successful transgenic lines for *NCED3* in Poplar. We obtained eight (8) independent OE lines and seven (7) independent RNAi lines (Supplementary Figure 2A). We tested all of these lines for *NCED3* expression under normal as well as after drought or water shortage conditions. Under normal conditions, all of the OE lines had significantly higher *NCED3* expression compared with WT. On the other hand, all of the RNAi lines had lower *NCED3* expression as compared with those in the WT plants (Supplementary Figure 2B). However, the expression profiling after 10, 20, and 30 min of the drought stress treatment showed the highest increase in NCED3 expression in the leaves of OE line #16, whereas the least change in NCED3 expression was recorded in RNAi line #26 (Supplementary Figure 2C). Therefore, we chose these two lines (OE line #16 and RNAi line #26) for further experiments.

According to the field and screen house-based drought stress experiments, the detached leaves of the RNAi lines exhibited severe curling and leaf rolling, which are characteristic of dehydration stress, after 10 and 20 min of detachment as compared with the leaves of the OE lines which showed no rolling and curling (Figure 8C). The PdNCED3 OE line was found to have a significantly tolerant phenotype under dehydration stress. These results were also supported by the significantly smaller stomatal apertures in the leaves of the NCED3 OE plants as compared with those of the WT and RNAi plants (Supplementary Figure 6B). Furthermore, water loss calculations indicated that the RNAi plants lost a significantly higher amount of moisture, whereas the OE plants lost a significantly lower amount of moisture when compared with the WT plants (Supplementary Figure 6C).

Furthermore, the RT-PCR analysis showed a significantly higher *PdNCED3* (*Potri.011G112400*) mRNA accumulation in the WT plants following the stress treatment with the highest expression after 30 min followed by 20 and 10 min of the treatment (Figure 8D). In the case of the OE line, the transcript accumulation of PdNCED3 was higher and faster than in the WT, whereas the RNAi line showed significantly lower and delayed expression of NCED3 (Figure 8D). Furthermore, the *PdNCED1* (*Potri.001G393800*) transcript accumulation was upregulated in both of the OE and RNAi lines after dehydration stress (Figure 8E), but there was no difference in its expression level when compared with the wild type. Among the ABA-signaling genes, the bZIP transcription factors *Potri.014g028200, Potri.002g125400*, and *Porti.004g140600* were ABA-responsive element-binding factors that enhance abiotic stress signaling (Zou et al., 2008; Hossain et al., 2010; Tang et al., 2012). Our results indicated that the expression of these three genes gradually increased in the order of OE, WT, and RNAi under dehydration stress (Figures 8F–H). Furthermore, *NCED3* OE resulted in a decrease in the expression of *Potri.010g248100, abscisic acid-insensitive 5-like protein 2* (*Pt-ABI2*), as compared with the RNAi and the WT line (Figure 8I).

### *PdNCED3* Overexpression Reduces Oxidative Damage and Increases ABA Content

**Figure 9:**
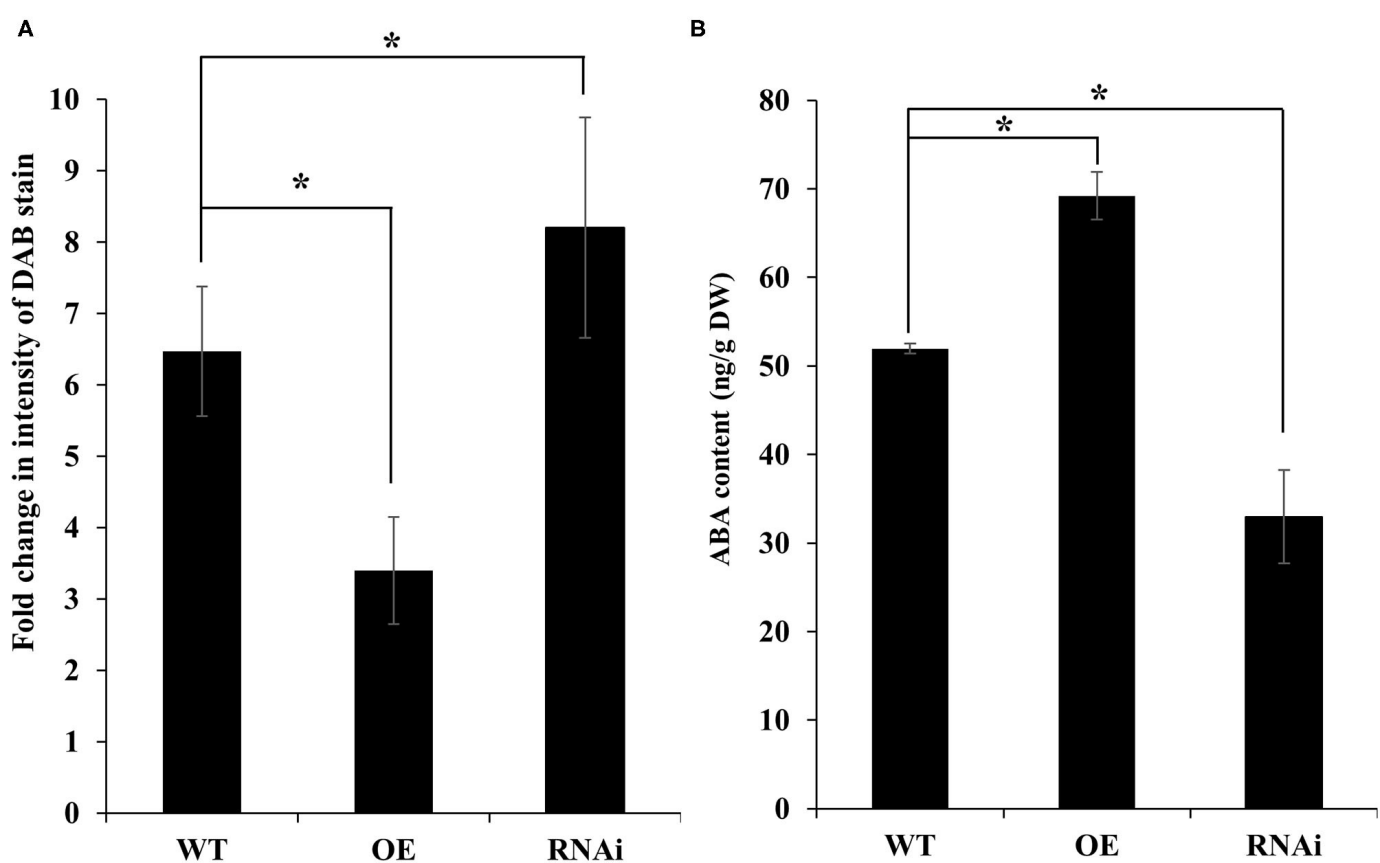
DAB staining and ABA contents in *PdNCED* transgenic plants. Diaminobenzidine staining in *NCED* transgenic plants **(A)**. ABA contents of *PdNCED* transgenic plants **(B)**. Data are presented as means of at least three replications. Error bars indicate standard error. Significant differences between means at *P* < 0.05 are indicated by asterisks (*).

To confirm that *PdNCED* reduces damage to plants by mitigating ROS-mediated oxidative damage, the amount of H_2_O_2_ accumulation in the leaves of the transgenic plants was assessed after 30 min of dehydration stress. As expected, leaves of the OE line showed significantly lower DAB stain intensity as compared with the WT and RNAi transgenic plants, indicating a much lower level of H_2_O_2_ production in the leaves of these plants following dehydration stress (Figure 9A).

It was worth knowing if the response of the *PdNCED3* OE and RNAi plants was related to the ABA contents in these plants. As expected, the ABA measurement results showed a significantly higher increase in the ABA contents of the OE line as compared with the RNAi plants (Figure 9B). These results were parallel with the expression patterns of *PdNCED3* in transgenic plants.

### Structural Differences in *P. davidiana* NCED Peptides

As described above, *PdNCED1* (*Potri.001G393800*) and *PdNCED3* (*Potri.011G112400*) were upregulated in the drought-tolerant cultivars but downregulated in the drought-sensitive cultivars. We aligned the sequence of these genes with their orthologous genes in *Populus trichocarpa* and found out that the *PdNCED* genes are unique in their sequence though both the genes showed more than 95% similarity to their respective *P. trichocarpa* orthologs. Single amino acid differences were found in the amino acid sequence of NCED3 *P. davidiana* and *P. trichocarpa* at seven different positions (Supplementary Figure 3). The first difference was a single amino acid difference at position 26 expressing isoleucine (I) in all *P. davidiana* cultivars compared with threonine (T) in *P. trichocarpa*. This was followed by another major difference between the NCED3 sequences of the two *Populus* species with the starting position number 55, where three consecutive proline threonine pairs (PTPTPT) were found in all the cultivars of *P. davidiana* as compared with only one pair in *P. trichocarpa* which may have a significant effect on the structure of this protein in the two species. Similarly, other *P. davidiana—P. trichocarpa* single amino acid differences were also found such as leucine (L66)—arginine (R66), arginine (R117)—glycine (G117), serine (S289)—threonine (T289), aspartic acid (D331)—asparagine (N), and alanine (A431)—proline (P431). Furthermore, the PdNCED3 peptide was found to be significantly smaller than PtNCED3. This was attributed to the multiple amino acid residues absent in the PdNCED3 sequence from the positions 298 to 330. This feature was especially prominent in the drought susceptible cultivars, Palgong 1 and Junguk 6–2. At position number 518, the NCED3 of both the drought-resistant *P. davidiana* cultivars and *P. trichocarpa* expressed a tyrosine (Y) residue, but it was absent in the NCED3 sequence of both the susceptible cultivars (Supplementary Figure 3).

Similarly, single and multiple amino acid differences were also found between the sequences of PdNCED1 and PtNCED1 (Supplementary Figure 4). Single-amino acid differences between *P. davidiana* and *P. trichocarpa* NCED1 were found at five different places. For example, at position number 48, proline (P) was found in all *P. davidiana* sequences but serine (S) in the *P. trichocarpa* sequence. Similarly, at position number 51 we found tyrosine (Y) in all the *P. davidiana* sequences as compared with cysteine (C) in *P. trichocarpa*. Another important difference that we observed was at position number 155 where we found valine (V) in all the *P. davidiana* sequences instead of leucine (L) in *P. trichocarpa*. We also found threonine (T) in all the *P. davidiana* sequences at position number 568 instead of asparagine (N) in *P. trichocarpa*.

With these differences in the sequence of NCED peptides from drought-resistant and susceptible cultivars, we wondered if these have any effect on the protein 3D structure. For this purpose, we submitted the deduced amino acid sequences to the I-Tasser server to predict the 3D structures. The results showed some interesting differences in the protein structure as well as the Co-factor/ligand binding (Supplementary Figure 5). PdNCED1 (Potri.001G393800) peptide was predicted to bind with an iron (Fe/Fe^2+^) co-factor only from the Palgong 2 and Seogwang 9 cultivars which are from the drought-tolerant group. Whereas, the PdNCED1 from the drought susceptible cultivars showed a tetraethylene glycol monooctyle ether (C8E) co-factor as a potential ligand. These structural differences may lead to the differential functioning of NCED in these plants resulting in significantly variable responses to drought stress.

## Discussion

Drought or water stress has long been considered a major limiting factor for crop production throughout the world. When human beings became able to differentiate between good, better, best, and bad; they selected only the best and fittest plants (and animals) for domestication. Drought or water stress resistance had been an important criterion for this selection and since then, one way or another; people have tried to select, introduce or develop crops that are resistant to water stress. However, drought is a natural phenomenon that is an integral part of climate and is certain to occur and therefore, must be expected. Since droughts are natural climatic phenomena and have been occurring for ages, plants have co-evolved with them, developing mechanisms to cope with water shortages during drought. These mechanisms are controlled by an ingenious transcriptional network of genes regulating complex biological processes to maximize plant performance under growth-limiting conditions of water stress. Based on several multi-location surveys and analyses, we identified four cultivars of *P. davidiana*. These four genotypes represent four different cultivars or varieties of poplar, widely grown in the Korean peninsula. Being different genotypes, their response to drought stress is also different. As shown in the results section, we tested the four genotypes for their response to drought stress based on morphological as well as physiological indicators and divided the genotypes into two groups i.e., tolerant group and susceptible group. The drought-resistant cultivars were Palgong 2 and Seogwang 9 whereas, the two drought susceptible cultivars were Palgong 1 and Junguk 6–2. The resistance and susceptibility of these cultivars were observed after subjecting whole plants to water stress as well as in the detached leaves experiment. Similar results were observed after the electron microscopy of the stomata following stress treatment. Water stress is often depicted by an increase in the accumulation of ROS which is why our susceptible cultivars, Palgong 2 and Junguk 6–2, accumulated significantly higher amounts of H_2_O_2_. Higher quantities of H_2_O_2_ and other ROS cause oxidative damage to plant tissues resulting in leaf deformation and curling as shown by the severe curling of leaves from the susceptible cultivars. In contrast, the leaves of the tolerant cultivars resisted leaf curling potentiated by their ability to keep their stomata almost closed to avoid the loss of water through evapotranspiration and loss of turgor. On the other hand, the leaves of the susceptible cultivars rapidly lost moisture and turgor pressure as their stomata were wide open. This is usually accompanied by a linear loss of fresh weight as shown in our results.

Plant responses to water stress are tightly regulated at the transcriptional level. The contrasting expression patterns of drought-specific genes in the tolerant and susceptible cultivars indicate the importance of these genes in regulating water stress responses in *P. davidiana* and other higher plants. Transcriptional responses subsequently dictate large-scale response pathways allocating resources and optimizing plant performance under stress through multiple routes. These include biological pathways regulating abiotic stress responses through the regulation of developmental phytohormones such as IAA, ABA, ethylene, GA, and defense hormones SA and JA (Chen et al., 2017). In this study, we found several genes involved in these hormonal pathways with changes in the expression by at least 2-folds following the stress treatment.

One of the most important and immediate responses of plants to water stress includes changes in the cellular redox state (Barchet et al., 2014). In plant cells, different compartments have different redox signatures which is the case for different types of cells at the tissue level. This differential redox signature is maintained through a complex transcriptional network of gene families including thioredoxin, peroxiredoxin, glutaredoxin, catalase, and dismutase genes (Mun et al., 2017a). We found multiple members of these families with differential expression patterns in the drought-resistant and susceptible cultivars. The transcriptional regulation of the above-mentioned pathways requires multiple transcription factor genes (WRKY, ERF, bZIP, MYB, and bHLH) which were abundantly and differentially expressed following the stress treatment. These results were similar to the findings reported in other studies involving drought stress-mediated transcriptomic analysis of *Populus* species such as Regier et al. (2009), Qiu et al. (2011), Raj et al. (2011), Peng et al. (2012), Shuai et al. (2014), Liu et al. (2015), Mun et al. (2017a), Mun et al. (2017b) and others.

The importance of ABA biosynthesis and homeostasis in plant responses to water stress is well-known as it positively regulates drought stress tolerance in plants. A key enzyme involved in ABA biosynthesis is NCED (Schwartz et al., 1997; Rook et al., 2001). The significantly higher expression of *PdNCED1* and *PdNCED3* and the concomitant increase in ABA accumulation enabled Palgong 2 and Seogwang 9 to tolerate water stress more than Palgong 1 and Junguk 6–2. These results were similar to the findings of Tan et al. (1997) who reported genetic evidence for the involvement of NCED in the ABA biosynthesis in maize under water stress conditions and Thompson et al. (2000b) who showed the involvement of *LeNCED1* in the ABA biosynthesis in tomatoes. The ABA-related role of NCED following drought stress is global and has been reported in several plant species (Burbidge et al., 1999; Qin and Zeevaart, 1999; Chernys and Zeevaart, 2000; Iuchi et al., 2000, 2001). This was confirmed by our studies on Arabidopsis *nced3* mutant plants as these plants failed to recover following 9 days of drought stress.

Over the course of transformation experiments in our laboratory (and elsewhere), it has been observed that the pure aspen *P. davidiana* is extremely difficult to transform genetically *via* tissue culture despite trying several variations and optimization in the methodology. Interestingly, *P. davidiana* has a significantly low vitality and rooting rate. More specifically, it is extremely difficult to obtain transgenic plants using *P. davidiana* leaves and stem and to our knowledge, there are no reported cases of successful transformations using *P. davidiana* leaves and/or stem. On the other hand, a successful genetic transformation has been reported using the aspen hybrid poplar “shanxin” from Northern China which is a cross between *P. davidiana* and *P. bollena* (Wang et al., 2011; Han et al., 2013; Ding et al., 2017; Han and Han, 2017). In our laboratory, we developed a shoot regeneration technology from the leaf and shoot derived calli of the hybrid poplar (*Populus alba* × *P. glandulosa*) (Choi et al., 2020) for the successful generation of transgenic plants using *Agrobacterium*-mediated transformation (Chung et al., 1989; Choi et al., 2005; Song et al., 2019). Using hybrid poplar, transgenic plants have been successfully generated with several different genes involved in a variety of physiological responses (Zhang et al., 2011; Shim et al., 2013; Choi et al., 2017; Yoon et al., 2018; Kim et al., 2020). Since the hybrid poplar used for the transformation in this study is an artificial cross between *P. alba* and *P. glandulosa*, we found that the *NCED3* DNA sequences of these two varieties were significantly similar to that of *P. davidiana*. The coding DNA sequence alignments indicated more than 99% of amino acid sequence similarity between *NCED3* from these three poplar varieties (Supplementary Figure 3). Hence, it is safe to use the hybrid poplar (*P. alba* × *P. glandulosa*) in the laboratory and field-based study instead of the pure aspen *P. davidiana*. The findings of our experiments on the transgenic plants confirmed the positive role of *NCED3* in the drought tolerance of forest trees such as Poplar.

All the drought-tolerant and susceptible cultivars used in this study expressed the two NCED genes, yet, the increase in the expression of these genes and the associated ABA quantities were significantly higher in the drought-tolerant cultivars than in susceptible cultivars. This could well be attributed to the differences in the amino acid sequence of these two genes between the tolerant and susceptible cultivars as shown in Supplementary Figures 3, 4. These sequence differences included single base pair differences at multiple places as well as long-chain differences right in the middle of the sequence especially for PdNCED3 (Potri.011G112400). Furthermore, the single base pair sequences involved amino acids such as cysteine and tyrosine residues which are active sites for post-translational modification by nitric oxide, i.e., S-nitrosylation at the cysteine residue and tyrosine nitration at the tyrosine residue. This indicates another possible explanation for the functional and expression differences between the two *NCED* genes of the tolerant and susceptible cultivars. Furthermore, these sequence differences had a great impact on the NCED 3D structure, especially that of PdNCED1 (Potri.001G393800). The ligand/co-factor binding prediction analysis showed the association of NCED1 with the Fe/Fe^2+^ co-factor in the drought-tolerant cultivars indicating the importance of Fe/Fe^2+^ co-factor for the normal functioning of the NCED. Contrastingly, PdNCED1 from the drought susceptible cultivars was found to be associated with the co-factor C8E. Furthermore, the sequence and structural differences among the NCED genes of *P. davidiana* and *P. trichocarpa* were also observed, indicating the relatively unique nature of the *P. davidiana* genome. Together, these results provide valuable information about the transcriptomic response of *P. davidiana* to water-limiting conditions and the specific role of NCED in regulating plant responses to water shortage. However, further investigation into the transcriptional control of drought-related responses, identification of key control genes, and functional genomics studies are suggested.

## Data Availability Statement

The datasets presented in this study can be found in online repositories. The names of the repository/repositories and accession number(s) can be found in the article/Supplementary Material.

## Author Contributions

S-UL, B-GM, E-KB, J-YK, and Y-IC performed the experiments. S-UL, J-YK, H-HK, and MS analyzed and curated the data. AH performed transcriptomic analysis. AH and B-WY wrote and reviewed the manuscript. B-WY provided the funds. All authors contributed to the article and approved the submitted version.

## Funding

This work was supported by the Forest Resource Genome Project (Grant Number S111415L070140), Korea Forest Research Institute, Republic of Korea.

## Conflict of Interest

The authors declare that the research was conducted in the absence of any commercial or financial relationships that could be construed as a potential conflict of interest.

## Publisher's Note

All claims expressed in this article are solely those of the authors and do not necessarily represent those of their affiliated organizations, or those of the publisher, the editors and the reviewers. Any product that may be evaluated in this article, or claim that may be made by its manufacturer, is not guaranteed or endorsed by the publisher.

## Supplementary Material

Supplementary Figure 1 Transcriptional differences between drought-tolerant and drought susceptible cultivars. The drought-related DEGs with statistical significance, at least two-fold change in their expression, and contrasting expression patterns in the drought and susceptible cultivars, following dehydration stress were identified. Different DEGs were expressed only in the drought-resistant cultivars but not in the susceptible cultivars. On the other hand, multiple DEGs were either downregulated in the drought-tolerant cultivars (downward blue arrows) but upregulated in the susceptible cultivars or *vice versa*.

Supplementary Figure 2 Independent PdNCED3 OE and RNAi transgenic lines. Eight (8) independent *PdNCED3* OE lines and seven (7) independent RNAi lines were obtained **(A)**. *NCED3* expression in the WT, RNAi, and OE lines under normal conditions **(B)**. *NCED3* expression in the WT, RNAi, and OE lines after 10, 20, and 30 min of drought stress treatment **(C)**.

Supplementary Figure 3 Amino acid sequence alignment of PdNCED3. Amino acid sequence alignment of PdNCED3 (Potri.011G112400) from the four cultivars under the study with NCED3 from *P. trichocarpa, P. glandulosa, Arabidopsis thaliana*, and the consensus sequence.

Supplementary Figure 4 Amino acid sequence alignment of PdNCED1. Amino acid sequence alignment of PdNCED1 (Potri.001G393800) from the four cultivars under the study with NCED1 from *P. trichocarpa, P. glandulosa*, and the consensus sequence.

Supplementary Figure 5 Predicted PdNCED1 3D structure and potential Co-factors. PdNCED1 (Potri.001G393800) peptide from the drought-tolerant cultivars, Palgong 2 and Seogwang 9 showed iron (Fe/Fe^2+^) as potential co-factor whereas, Palgong 1 and Junguk 6–2 showed tetraethylene glycol monooctyle ether (C8E) as a potential co-factor ligand. The predicted 3D structure, associated co-factor, and ligand binding sites information with the highest C-Score were selected.

Supplementary Figure 6 Stomatal aperture and water loss measurements. Measurements of stomatal aperture in the leaves of the drought-tolerant and drought susceptible *P. davidiana* cultivars **(A)**. Measurements of stomatal aperture in the leaves of the *P. davidiana* WT, OE, and RNAi lines **(B)**. Water loss measurements from the leaves of *P. davidiana* WT, OE, and RNAi lines **(C)**.

Supplementary Figure 7 Generation of transgenic Poplar plants via tissue culture.

Supplementary Table 1 List of primers used for PCR.
