# Supplementary figures and images for: Drought Stress-Mediated Transcriptome Profile Reveals NCED as a Key Player Modulating Drought Tolerance in Populus davidiana
Source: Front Plant Sci. 2021 Oct 28;12:755539. doi: 10.3389/fpls.2021.755539 (PMC8581814; doi:10.3389/fpls.2021.755539)

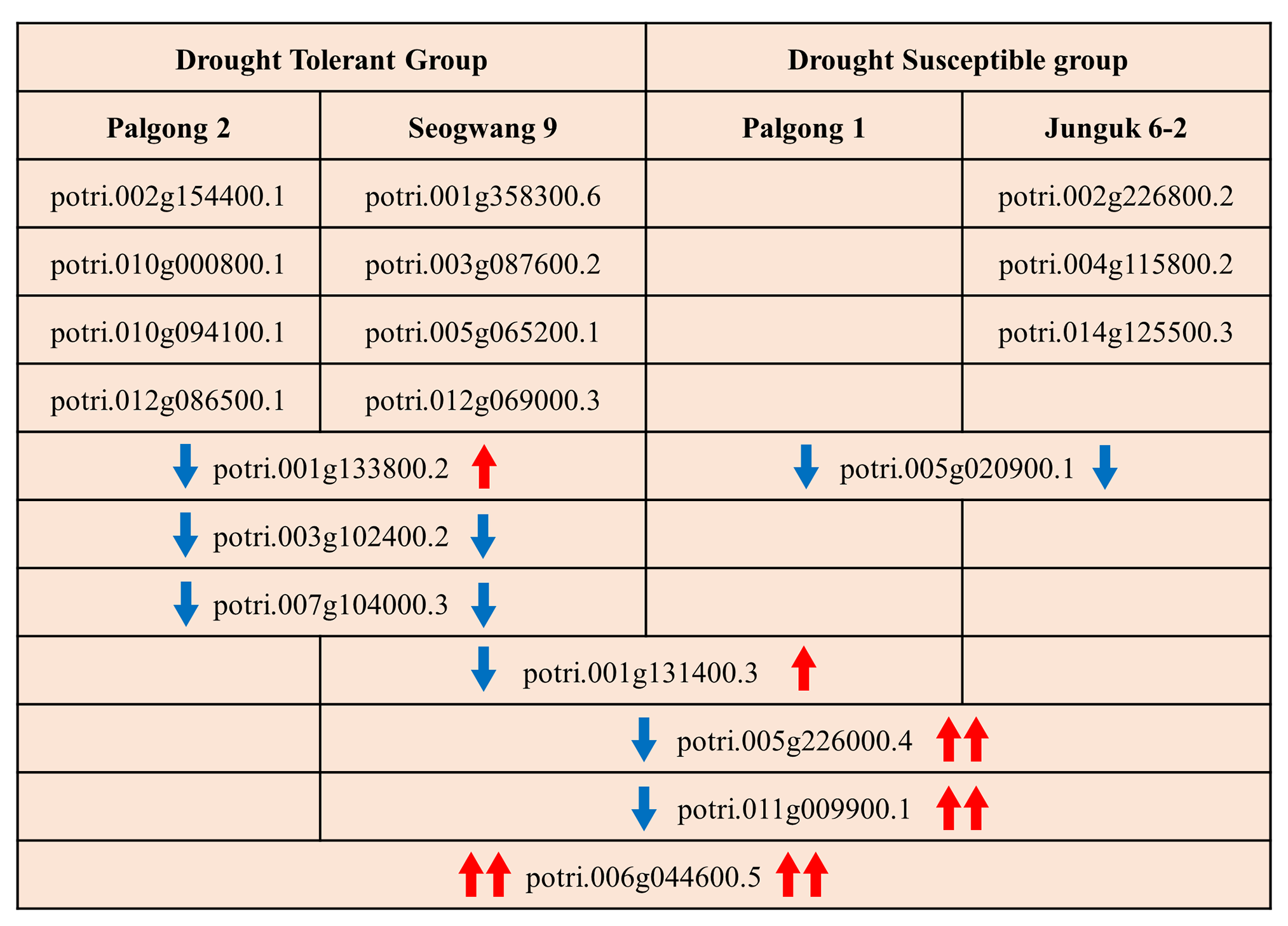

Supplement: Supplementary Figure 1 — Transcriptional differences between drought-tolerant and drought susceptible cultivars. The drought-related DEGs with statistical significance, at least two-fold change in their expression, and contrasting expression patterns in the drought and susceptible cultivars, following dehydration stress were identified. Different DEGs were expressed only in the drought-resistant cultivars but not in the susceptible cultivars. On the other hand, multiple DEGs were either downregulated in the drought-tolerant cultivars (downward blue arrows) but upregulated in the susceptible cultivars or vice versa. [file Image_1.TIF]

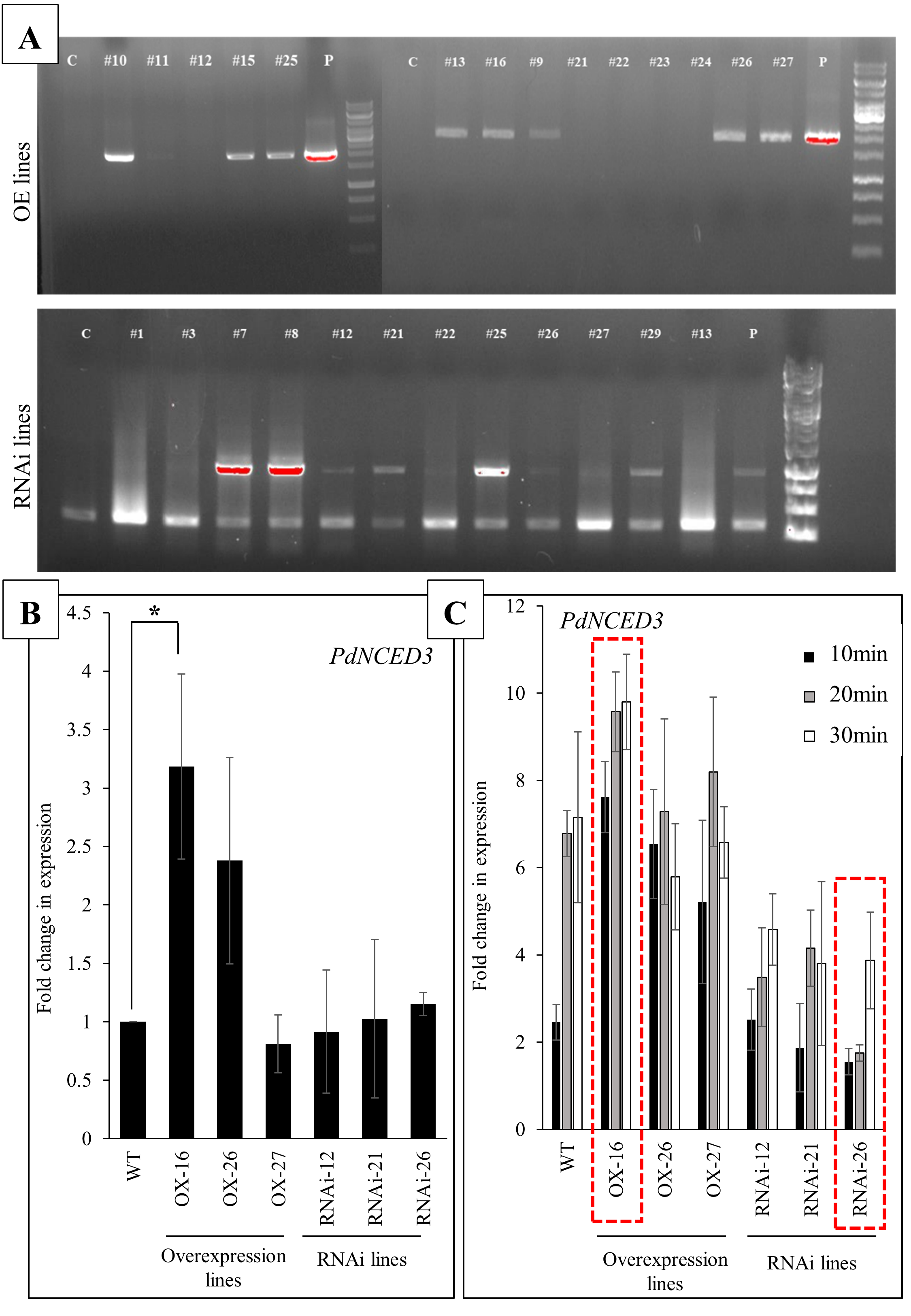

Supplement: Supplementary Figure 2 — Independent PdNCED3 OE and RNAi transgenic lines. Eight (8) independent PdNCED3 OE lines and seven (7) independent RNAi lines were obtained (A). NCED3 expression in the WT, RNAi, and OE lines under normal conditions (B). NCED3 expression in the WT, RNAi, and OE lines after 10, 20, and 30 min of drought stress treatment (C). [file Image_2.TIF]

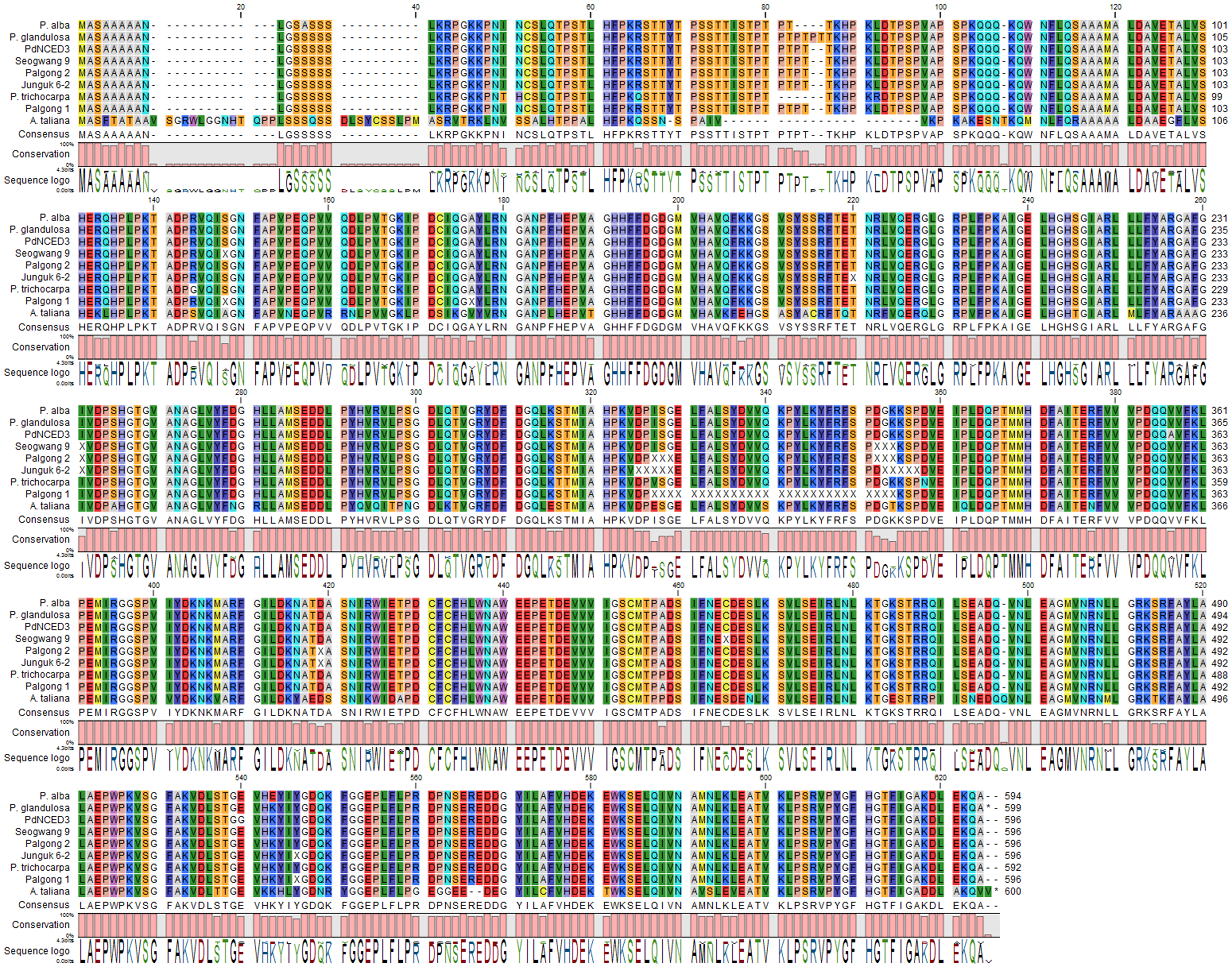

Supplement: Supplementary Figure 3 — Amino acid sequence alignment of PdNCED3. Amino acid sequence alignment of PdNCED3 (Potri.011G112400) from the four cultivars under the study with NCED3 from P. trichocarpa, P. glandulosa, Arabidopsis thaliana, and the consensus sequence. [file Image_3.TIF]

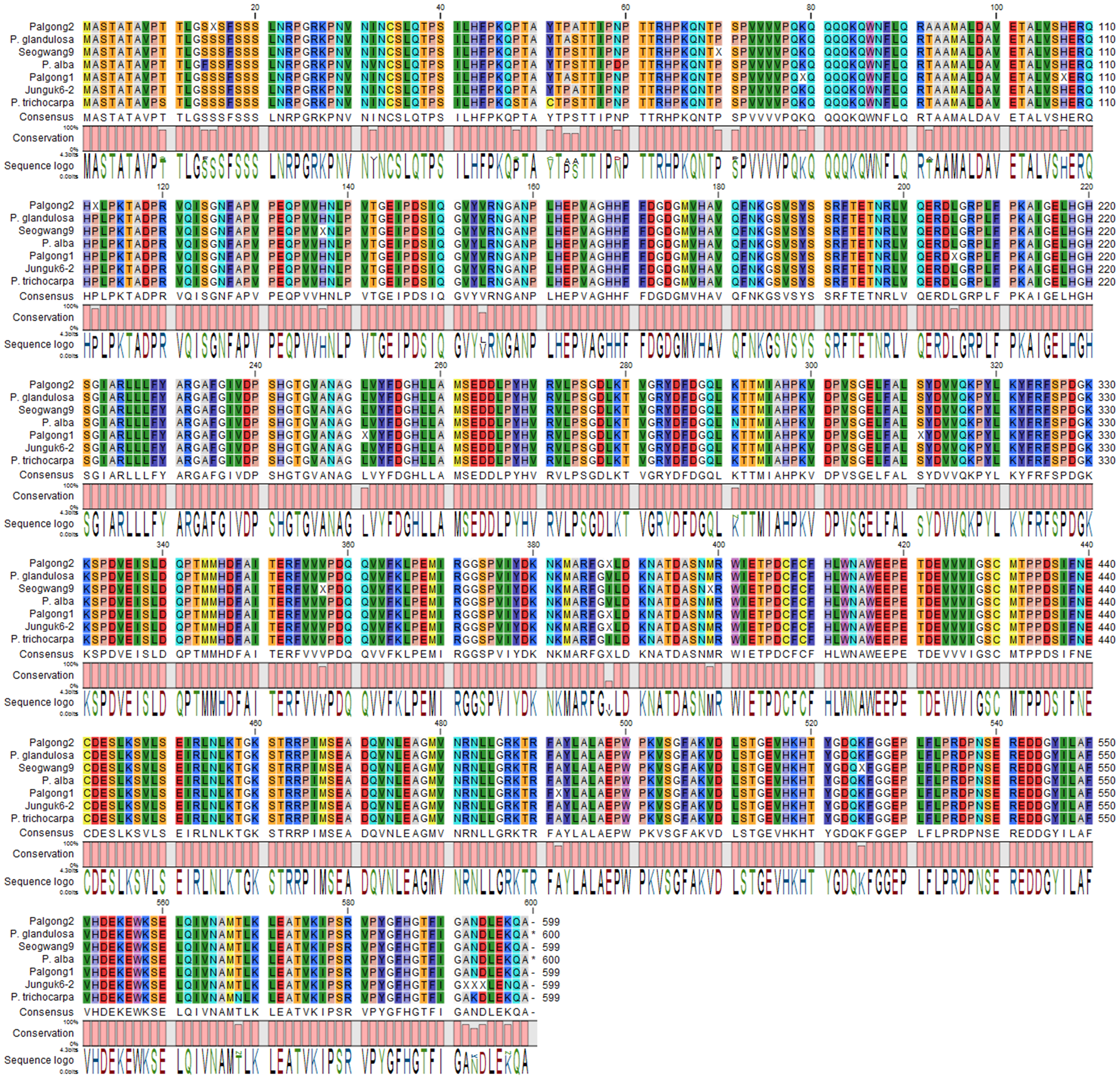

Supplement: Supplementary Figure 4 — Amino acid sequence alignment of PdNCED1. Amino acid sequence alignment of PdNCED1 (Potri.001G393800) from the four cultivars under the study with NCED1 from P. trichocarpa, P. glandulosa, and the consensus sequence. [file Image_4.TIF]

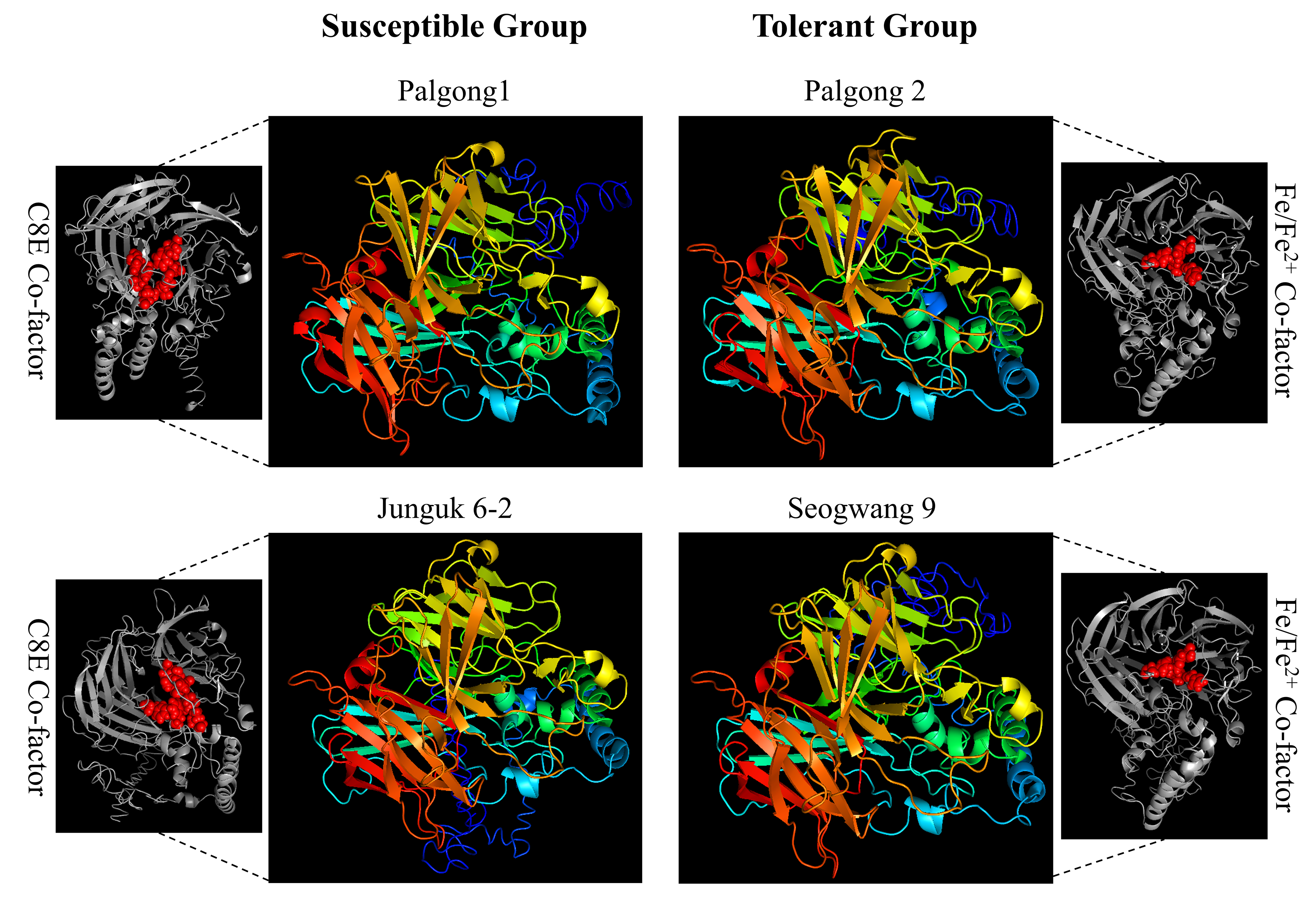

Supplement: Supplementary Figure 5 — Predicted PdNCED1 3D structure and potential Co-factors. PdNCED1 (Potri.001G393800) peptide from the drought-tolerant cultivars, Palgong 2 and Seogwang 9 showed iron (Fe/Fe2+) as potential co-factor whereas, Palgong 1 and Junguk 6–2 showed tetraethylene glycol monooctyle ether (C8E) as a potential co-factor ligand. The predicted 3D structure, associated co-factor, and ligand binding sites information with the highest C-Score were selected. [file Image_5.TIF]

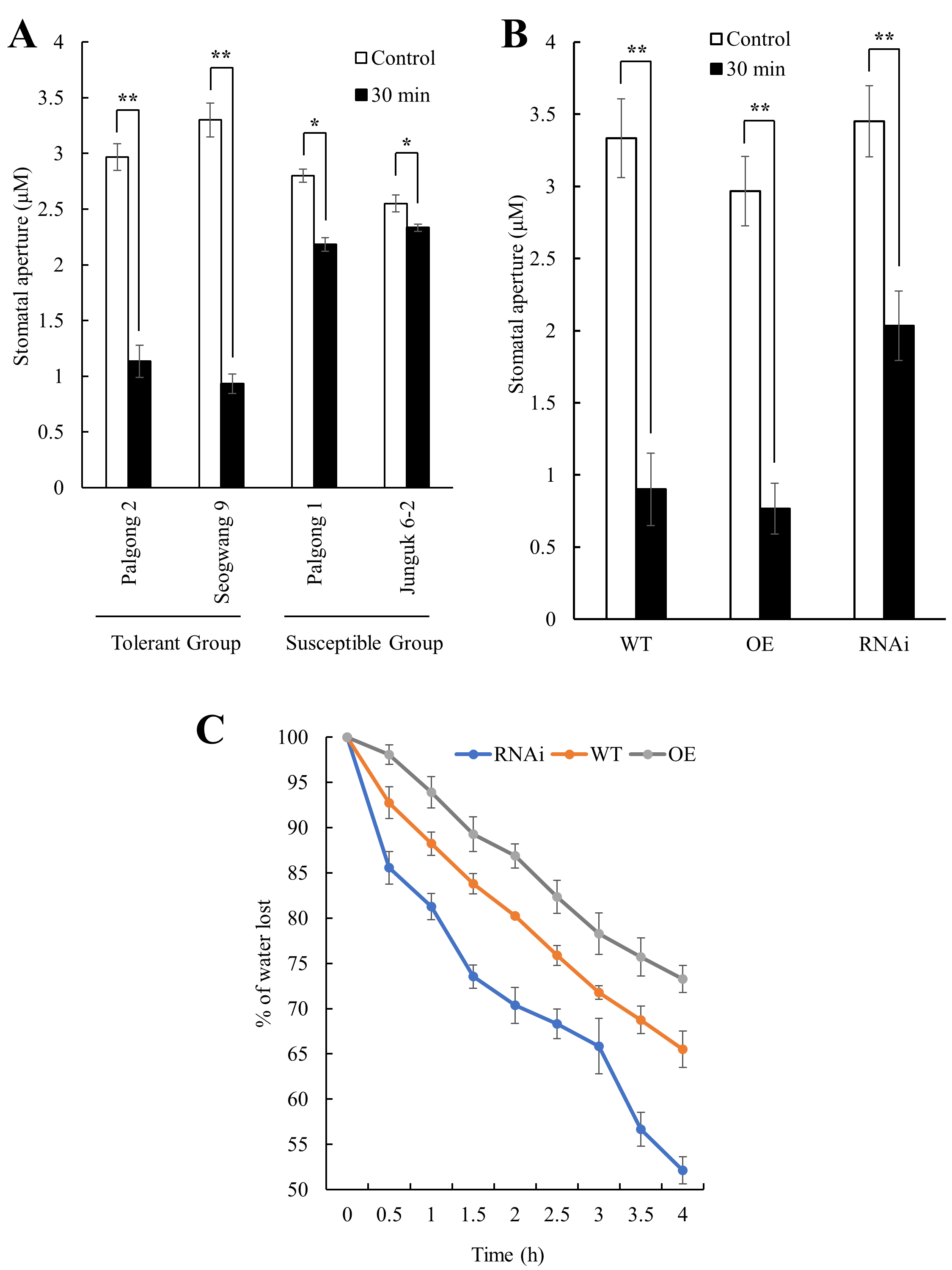

Supplement: Supplementary Figure 6 — Stomatal aperture and water loss measurements. Measurements of stomatal aperture in the leaves of the drought-tolerant and drought susceptible P. davidiana cultivars (A). Measurements of stomatal aperture in the leaves of the P. davidiana WT, OE, and RNAi lines (B). Water loss measurements from the leaves of P. davidiana WT, OE, and RNAi lines (C). [file Image_6.TIF]

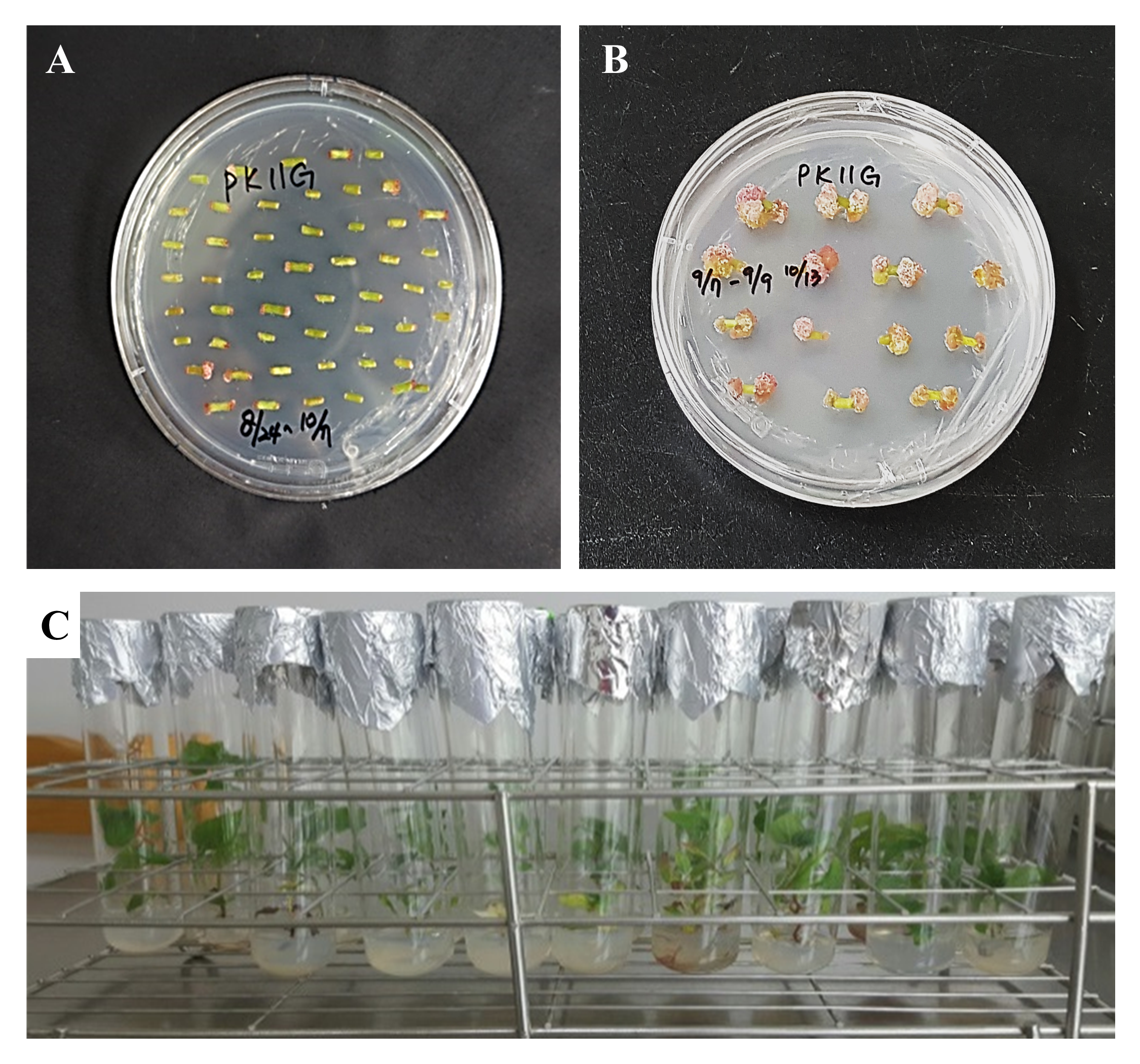

Supplement: Supplementary Figure 7 — Generation of transgenic Poplar plants via tissue culture. [file Image_7.TIF]
