# Supplementary material for: Drought Stress-Mediated Transcriptome Profile Reveals NCED as a Key Player Modulating Drought Tolerance in Populus davidiana
Source: Front Plant Sci. 2021 Oct 28;12:755539. doi: 10.3389/fpls.2021.755539 (PMC8581814; doi:10.3389/fpls.2021.755539)
Supplement: Supplementary Table 1 — List of primers used for PCR. [file Table_1.DOCX]

**Supplementary Table S1. List of primers used for PCR.**

| **S. No.** | **Gene Name/Accession Number** | **Forward primer sequence (5′ – 3′)** | **Reverse primer sequence (5′ – 3′)** |
| --- | --- | --- | --- |
| **qRT-PCR primers** | | | |
| **1** | Potri.011G112400 | AGAAACGGAGCCAATCCATTT | CCCACGTTCTTGAACAAGCC |
| **2** | Potri.001G393800 | ACCCAAAACAAAACACGCCC | AAGGGTGCTGACGTTCATGT |
| **3** | Potri.004G140600 | ATCACCGGATGTGATTGCGA | CAAGGCCCTGTCAATGTCCT |
| **4** | Potri.014G028200 | AACCTCTGCTGGGGTACAAG | TCCCACACCCCTATTCTGCT |
| **5** | Potri.002G125400 | TGTGACGCAGCCTAATAACGA | CTCCCAATCCAACCACTCCC |
| **6** | Potri.003G165500 | GTGTTTTCCCCCTTTACCTTCC | TATTGCTTTCGCCGTCAGGT |
| **7** | Potri.010G248100 | CTGCCCAGACTACAGCTTTG | GTCCACGGATGCTTCAGCTA |
| **8** | Potri.017G152900 | TCATCCTGGTGCAGGAACAC | CCTGCAACAGCACCAGTTTC |
| **9** | Potri.001G309500 (Actin) | TTCTACAAGTGCTTTGATGGTGAGTTC | CTATTCGATACATAGAAGATCAGAATGTTC |
| **Transgenic vector construction by Gateway system** | | | |
| **10** | Potri.011G112400_pK2_attB1/attB2 | AAAAAGCAGGCTATGGCTTCAGCAGCAGC  (pK2_11G_attB1 F) | AGAAAGCTGGGTCTAGGCCTGTTTCTCC  (pK2_11G_attB2_R) |
| **11** | Potri.011G112400_pK7_attB2/attB1 | AGAAAGCTGGGTATGGCTTCAGCAGCAGCAGCA (pK7_11G_attB2 F) | AAAAAGCAGGCTTCTAGTTTTGGGTGCTTGGT (pK7_11G_attB1 R) |
| **Genotyping primers** | | | |
| **12** | Potri.011G112400 Overexpression | CGCAAGACCCTTCCTCTATATAAG  (pK2_35S_Pro F) | TTGTACAAGAAAGCTGGGTCTAG  (pK2_11G_OX R) |
| **13** | Potri.011G112400 RNAi | CACATGAGCGAAACCCTATAAGA (pK7GWIG2(1) T35S_nos F) | AAGATGCCTCTGCCGACAGTGGTC  (pK7GWIG2(1) 35S_Pro R) |
